# Topology engineering of COFs *via* localized 1D–2D unit interconnection to facilitate interfacial electron transfer for efficient gold recovery from e-waste leachates

**DOI:** 10.1039/d5sc05206h

**Published:** 2025-11-07

**Authors:** Jiaxing Xiong, Qi An, Hao Xiang, Yu Zhou, Yuan Zhang, WenJing Chen, Boxian Ren, Shixiong Wang, Huiping Bai, Hong Guo, Xiangjun Yang

**Affiliations:** a School of Chemical Science and Technology, Yunnan University Kunming 650091 China; b International Joint Research Center for Advanced Energy Materials of Yunnan Province, School of Materials and Energy, Yunnan University Kunming 650091 China

## Abstract

Developing cost-effective adsorbents for gold recovery from electronic waste (e-waste) is crucial. Here, we report a dimensional engineering strategy to fabricate covalent organic framework (COF) heterostructures with locally interconnected dimensions, enabling highly efficient host–guest recognition of Au(iii) in e-waste leachates. The heterostructures feature local interconnections between 2D and 1D units, introducing additional electron transport pathways. This structural innovation optimizes the electron transport kinetics essential for adsorptive redox reactions. Experimental results demonstrate that the locally interconnected dimensional architecture of TpaIda-2DCOF achieves a static saturation adsorption capacity of 3601 mg g^−1^ for Au(iii) among the highest reported, which reaches an unprecedented 6900 mg g^−1^ under ultrasonic assistance. A fixed-bed system processes 35 L of e-waste leachate, yielding 23.98 karat gold *via in situ* reduction while retaining a material cost below 10 CNY g^−1^—establishing it as one of the most cost-effective COF-based adsorbents reported to date. Theoretical calculations and spectral analyses reveal that the additional charge transport channels created by the heterostructure facilitate the directional conversion of Au(iii) to Au(0) through a stepwise single-electron reduction mechanism, thus constructing a closed-loop process for gold capture and recovery.

## Abstract 2

The establishment of local dimensional interconnectivity creates novel interlayer charge transport channels, which significantly improves electron transport efficiency and achieves highly selective gold recovery.

## Graphical abstract

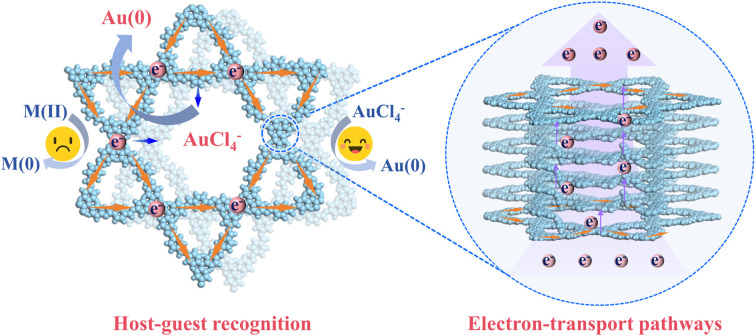

## Introduction

With rapid technological advancements and the shortening of product lifecycles, waste electrical and electronic equipment has emerged as an “urban mine” rich in recoverable gold resources.^1–3^ Meanwhile, the escalating demand for gold has made efficient targeted recovery from secondary resources increasingly imperative. Although biomass, metal–organic frameworks, activated carbon, and other materials have been extensively explored for gold recovery,^4–8^ they commonly suffer from limitations such as modest adsorption capacity, sluggish kinetics, high synthesis costs, and suboptimal selectivity. A key challenge in most contemporary designs is that adsorbed gold species is partially present in the ionic state, a phenomenon that significantly amplifies the complexity of subsequent recovery processes. These limitations are primarily attributed to the insufficient electron-donating capacity of redox-active sites within the adsorbent host and inefficient electron transfer processes, which hinder the directional conversion of Au(iii) to Au(0). Additionally, the high synthesis costs of materials render them dependent on multiple cycles of regeneration to achieve economic viability. This twofold bottleneck motivates a critical scientific question: can we design inexpensive materials with intrinsic electron-transport pathways to construct an integrated system for concurrent adsorption-enrichment and *in situ* reduction, thus achieving a capture and recovery closed-loop process?

Covalent organic frameworks^9–11^ (COFs) emerge as ideal material candidates owing to their tunable functionalities, efficient electron/ion mobility,^12^ and designable redox sites.^13^ However, despite the implementation of strategies such as pre-synthetic modification,^14^ post-synthetic functionalization,^15–18^ and photocatalysis^19^ for Au(iii) recovery in recent years, the recovery efficiency remains constrained by inefficient electron transfer processes. Thus, the construction of efficient electron transport channels to boost the electron transfer kinetics and thereby enable efficient Au(iii) enrichment and recovery represents a central challenge in this field. Notably, the precise and predictable synthesis of COFs with tunable dimensions^20–22^ and optimized electron transfer efficiency *via* isomer-based synergistic regulation strategies^23^ offers a promising pathway for designing high-efficiency Au(iii) recovery systems incorporating host–guest recognition functionalities. The reason isomers can build COFs with different structural dimensions is that linear building blocks experience fewer spatial constraints during COF formation, allowing them to propagate easily along the edges to the entire 2D planar frame. In contrast, nonlinear linkers tend to exhibit non-edge transmission of COF topologies due to the inherent angular limitations of their reactive sites, leading to the formation of specific banded frameworks. Based on the above structural characteristics, electron transport pathways exist in the in-plane conjugated structures of 2D COFs and ribbon-like frameworks of 1D COFs. Due to differences in the conjugation degree and van der Waals gaps governing in-plane charge transport and inter-ribbon charge transport, distinct variations in charge migration resistance emerge between these two transport mechanisms. Although isomers have been used to construct COFs with different structural dimensions, no studies have yet explored the impact of structural dimensionality on Au(iii) recovery performance. At the same time, the structure–property relationships and transport mechanisms of electron-transport pathways in different dimensions remain unclear. Therefore, there is an urgent need to establish efficient electron-transport channels through dimensional regulation strategies and construct an *in situ* capture and recovery closed-loop system for Au(iii) to meet the practical requirements of resource recovery applications.

**Scheme 1:**
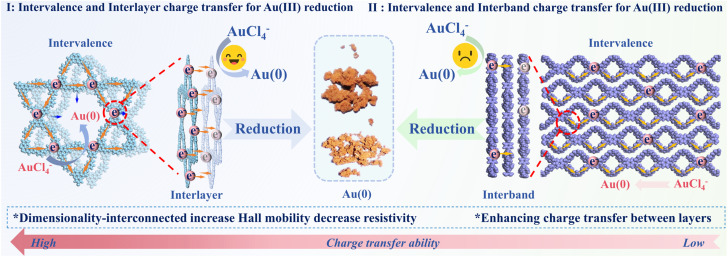
Schematic diagram of the process of intervalence charge transfer and interlayer charge transfer promoting Au(iii) reduction.

Herein, we introduce a dimensional engineering strategy, leveraging isomer-modulated local 1D–2D unit interconnections within COFs, to construct a closed-loop Au(iii) capture and *in situ* recovery system. This approach, realized *via* a facile three-component, one-pot synthesis, aims to overcome electron transfer limitations inherent in conventional COF adsorbents. We demonstrate that these tailored hetero-dimensional COFs, exemplified by TpaIda-2DCOF, exhibit significantly enhanced electron transport properties, evidenced by an increase in Hall mobility to 1.59 cm^2^ V^−1^ s^−1^ and a 4.7 × 10^4^ Ω cm decrease in resistivity. These improvements, attributed to the newly formed electron transfer pathways at the 1D–2D interfaces (Scheme 1), directly translate to superior Au(iii) reduction kinetics and an exceptional static adsorption capacity. We used a variety of structural characterization methods to confirm the authenticity of the structure. And the stepwise reduction mechanism of Au(iii) was analyzed through theoretical calculation and spectral analysis. This work not only presents a highly cost-effective COF adsorbent for gold recovery but also offers a novel paradigm for rationally designing COF architectures with precisely controlled electronic properties for advanced applications.

## Results and discussion

### Material preparation and characterization

**Fig. 1:**
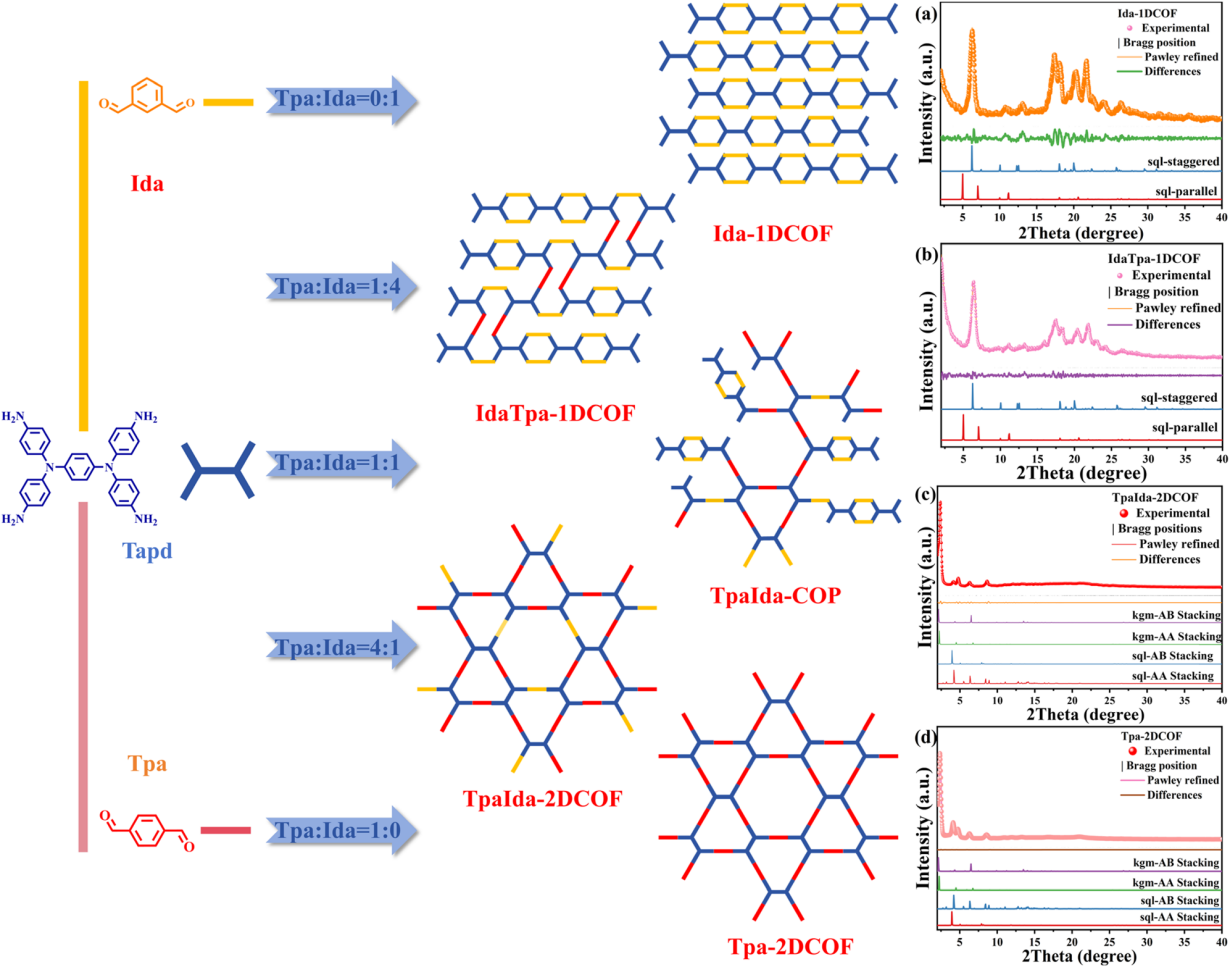
Synthesis of Tpa-2DCOF, Ida-1DCOF, TpaIda-2DCOF, and IdaTpa-1DCOF from Tpda, Ida, and Tpa building units. (Note: this process is only a schematic diagram of possible structures and does not represent the actual structure. Local dimensional distinctions are emphasized.) (a and b) PXRD patterns of synthesized Ida-1DCOF, IdaTpa-1DCOF and simulated Ida-1DCOF, IdaTpa-1DCOF sql-staggered and sql-parallel stacking modes; (c and d) PXRD patterns of synthesized TpaIda-2DCOF, Tpa-2DCOF and simulated TpaIda-2DCOF, Tpa-2DCOF sql-AA, sql-AB, kgm-AA, and kgm-AB stacking modes.

To achieve local dimensional control of the COFs, this study utilized both linear and non-linear connectors as building units. The COFs were synthesized under solvothermal conditions (details in the SI). Specifically, terephthalaldehyde (Tpa), isophthalaldehyde (Ida), and *N*,*N*,*N*′,*N*′-tetrakis(4-aminophenyl)-1,4-benzenediamine (Tapd) were used as precursors, with acetic acid serving as the catalyst. The reaction was conducted at 120 °C for 72 h in *n*-butanol/*o*-dichlorobenzene mixed solvents, followed by Soxhlet extraction for 24 h using acetone and tetrahydrofuran. To ensure that the COFs have excellent crystallinity and high specific surface areas, *n*-hexane (a solvent with low surface tension) was used for washing for 24 h. By adjusting the isomer ratios, a series of COFs with tunable electron transport efficiency and local dimensional distinctions were synthesized (Fig. 1).

In accordance with reticular chemistry principles, combining Tapd (a *D*_2h_-symmetric monomer) and Tpa (a linear *C*_2_-symmetric monomer) results in two distinct crystalline network topologies: kagome (kgm) networks with triangular and hexagonal pores, and square (sql) networks with uniform rhombus-shaped pores. These networks can adopt either eclipsed (AA) or staggered (AB) stacking modes, leading to four configurations: kgm-AA, kgm-AB, sql-AA, and sql-AB. When paired with Ida (a nonlinear *C*_2_-symmetric linker), Tpda forms 1D ribbon-like structures with a 4-c **sql** topology, which can theoretically exist in parallel (4c-sql-parallel) or staggered (4c-sql-staggered) stacking modes. To determine the structures of the COFs, experimental powder X-ray diffraction (PXRD) patterns were simulated using the Reflex module in BIOVIA Materials Studio software. The PXRD patterns of Ida-1DCOF matched the 4c-sql-staggered model, confirming a staggered 1D ribbon structure (Fig. 1a). In contrast, the diffraction patterns for Tpa-2DCOF aligned with the kgm-AA model, validating a 2D dual-pore structure with eclipsed stacking (Fig. 1d). Notably, systems with Ida/Tpa molar ratios less than 1 adopted the kgm-AA model, while ratios greater than 1 caused a structural transition to the 4c-sql-staggered model (Fig. 1b and c). This indicates that limited isomer incorporation resulted in changes in localized connectivity without altering the overall topology. This may be due to an excess of one monomer establishing a “dominant assembly guidance” in the system, directing the directional arrangement of a small number of other monomers through its spatial configuration, and reducing reactive site mismatch, ultimately improving crystallinity significantly. However, when the molar ratio of Ida to Tpa is near-equimolar, their reactive sites undergo nearly equivalent competitive reactions. At this point, the system lacks guidance for “directional growth,” causing crystal growth to terminate due to disordered assembly and ultimately yielding an amorphous TpaIda-COP (Fig. S1), consistent with previous findings.^24^ Overall, these results demonstrate that larger disparities in the isomeric ratio were associated with higher crystallinity, whereas near-balanced ratios hindered the formation of highly crystalline structures (Fig. S2). The experimental PXRD data for Tpa-2DCOF and TpaIda-2DCOF closely aligned with their respective Pawley-refined structural models, yielding *R*_wp_ values of 0.49% and 2.93%, and *R*_p_ values of 0.27% and 1.35%, respectively. In contrast, Ida-1DCOF and IdaTpa-1DCOF exhibited slightly lower fitting accuracy, with *R*_wp_ < 10.00% and *R*_p_ < 5.00%, yet still confirming structural consistency with the simulated unit cell parameters. The strongest diffraction peak of Tpa-2DCOF appeared at 2.2° corresponding to the (100) plane reflection of its hexagonal structure, while additional peaks at 4.2°, 4.8°, 6.7°, and 8.7° were attributed to the (110), (200), (210) and (310) planes, respectively. Notably, within the 2*θ* range of 1.0–10.0°, the strongest diffraction peak of TpaIda-2DCOF migrates slightly compared to Tpa-2DCOF (Fig. S3), suggesting a minor variation in the pore size between their hexagonal pore structures.

**Fig. 2:**
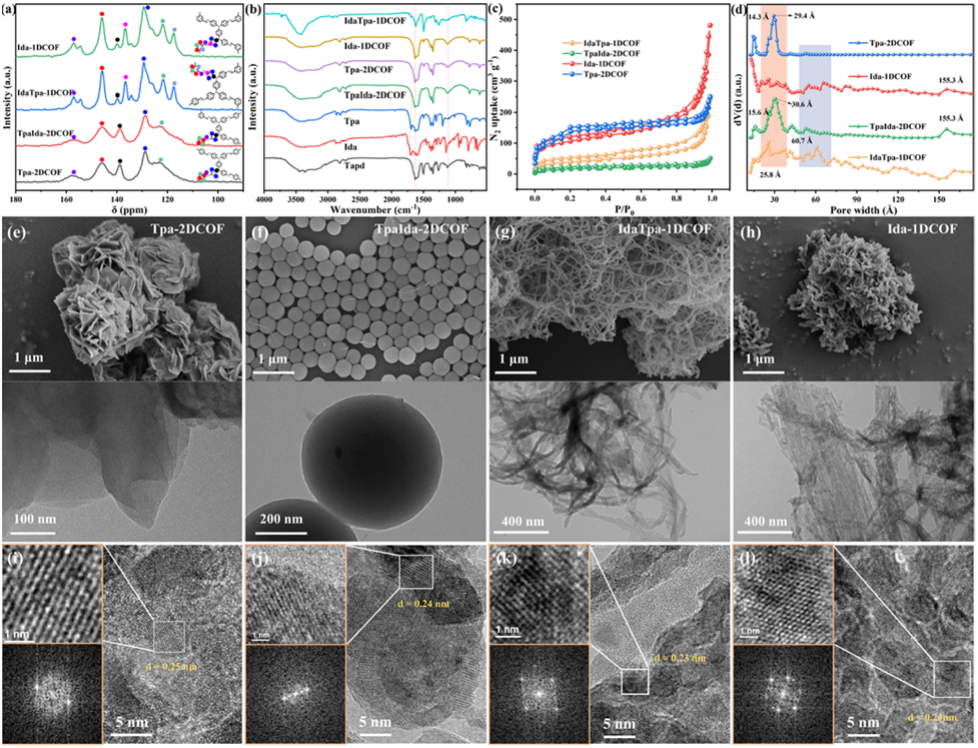
(a) ^13^C CP-MAS NMR spectra, (b) FT-IR spectra, (c) N_2_ adsorption curves at 77 K and (d) pore distribution curves of Tpa-2DCOF, TpaIda-2DCOF, IdaTpa-1DCOF, and Ida-1DCOF. The SEM and TEM images of (e) Tpa-2DCOF, (f) TpaIda-2DCOF, (g) IdaTpa-1DCOF, and (h) Ida-1DCOF. HRTEM images (right), clear lattice fringes at high magnification (top left) and the corresponding FFT pattern (bottom left) of (i) Tpa-2DCOF, (j) TpaIda-2DCOF, (k) IdaTpa-1DCOF, and (l) Ida-1DCOF.

The formation of imine linkage frameworks was further certified by solid state ^13^C cross-polarization magic-angle-spinning (CP-MAS) NMR spectroscopy which showed a peak at 167 ppm for the carbon of the imine with other overlapping peaks at 115–160 ppm for aromatic carbons in the structure (Fig. 2a). Fourier transform infrared (FTIR) spectra (Fig. 2b) further showed that, the C=O peak at 1698 cm^−1^ belonging to Tpa or Ida decreased obviously for the four COFs, while a new peak emerged at 1623 cm^−1^, indicating the complete conversion of the C=O bond to C=N bond.^25,26^ X-ray photoelectron spectroscopy (XPS) N 1s spectra confirmed the formation of the C=N bonds (Fig. S4), and elemental analysis results showed that the contents of N, H, and C were consistent with the theoretical values (Table S1), further verifying the structural accuracy.

The porosities and specific surface area of the four COFs were characterized using N_2_ sorption isotherms at 77 K. Fig. 2c reveals that the Brunauer–Emmett–Teller (BET) surface areas of Tpa-2DCOF and Ida-1DCOF were 471.8 and 378.1 m^2^ g^−1^, respectively. Non-local density functional theory (NLDFT) analysis indicates that the pore distributions of TpaIda-2DCOF and IdaTpa-1DCOF, synthesized *via* the three-component strategy, closely resembled those of Tpa-2DCOF and Ida-1DCOF across various pore width ranges (Fig. 2d). This suggests that the introduction of local dimensional distinctions altered the pore size distribution of the traditional **kgm** topology network, resulting in the formation of a hierarchical pore structure with both micropores and mesopores. Notably, the BET surface area of TpaIda-2DCOF was reduced, likely due to the intercalation of mixed monomers during COF formation, which partially obstructed the porous channels. Additionally, the larger nanosphere size of TpaIda-2DCOF, compared to the nanosheets of Tpa-2DCOF (Fig. 2e–h), may also contribute to the decrease in the specific surface area.

The morphology of the COFs was examined *via* a transmission electron microscope (TEM) and scanning electron microscope. Tpa-2DCOF displayed nanosheets with a rosebud-like structure that intergrew to form larger spherical aggregates approximately 1 µm in size (Fig. 2e). In contrast, TpaIda-2DCOF exhibited a spherical morphology (Fig. 2f). The scanning electron microscope (SEM) images of Ida-1DCOF exhibited a rigid nanowire structure, while IdaTpa-2DCOF displayed a flexible nanoribbon structure (Fig. 2g), with a significantly longer length compared to Ida-1DCOF (Fig. 2h). These morphological differences indicate that TpaIda-2DCOF and IdaTpa-1DCOF were not simple physical blends but rather COFs formed through the combined participation of two isomers. The changes in morphology were likely due to the synergistic effect of the isomers in COF formation. Furthermore, TEM images confirmed the morphological features observed in SEM. These findings (Fig. S5) highlight the potential of isomeric modulation in controlling the COF structure and morphology. High-resolution transmission electron microscopy (HRTEM) was employed to investigate the crystalline structure of the four COFs. The results show that the interplanar spacing observed for Tpa-2DCOF is 0.25 nm (Fig. 2i), corresponding to the (310) plane, which is consistent with previous literature reports.^27^ For TpaIda-2DCOF, well-defined lattice fringes with an interplanar spacing of 0.24 nm were observed (Fig. 2j). This observed spacing correlates well with the distinct features in its PXRD pattern, and this difference can be attributed to the unique local interconnections between its 2D and 1D units. Similarly, clear lattice fringes with identical interplanar spacing were also observed in Ida-1DCOF and IdaTpa-1DCOF (Fig. 2k and l), suggesting that they likely belong to the same crystal plane. In summary, the well-resolved lattice fringes in HRTEM images, along with the sharp diffraction spots in the corresponding fast Fourier transform (FFT) patterns, collectively demonstrate the high crystallinity of the four COFs.

**Fig. 3:**
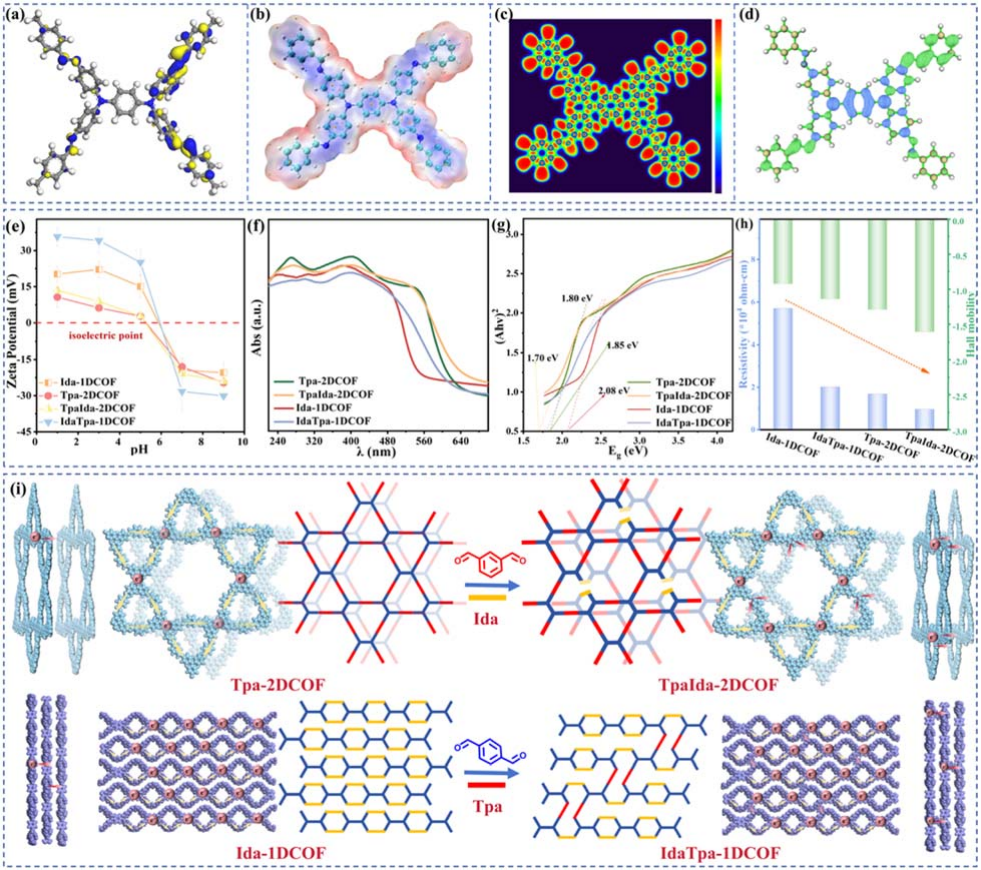
(a) The LUMO, (b) ESP, (c) ELF mapping, and (d) hole & electron diagram of a TpaIda-2DCOF unit. (Note: only molecular fragments were calculated, and complete periodic COF structures were not considered.) The zeta potential values (e), UV-vis DRS spectra (f), (*Ahv*)^2^–*hv* relationship curve (g), and Hall mobility and resistivity (h) of Ida-1DCOF, Tpa-2DCOF, TpaIda-2DCOF, and IdaTpa-1DCOF. (i)Schematic diagram of the formation of local dimensional distinctions and a schematic diagram of electron transport within and between layers. (Note: this process is only a schematic diagram of possible structures and does not represent the actual structure. Local dimensional distinctions are emphasized).

To understand the electron transfer mechanism, wave function analysis was performed using Multiwfn 3.8(dev).^28^ The delocalization of the lowest unoccupied molecular orbital (LUMO) across the molecular framework (Fig. 3a) indicated superior charge transfer capability, which was critical for promoting the recognition and reduction of Au(iii). Furthermore, electrostatic potential (ESP) mapping (Fig. 3b) revealed that C=N sites exhibited significantly higher charge density than the C–N bond, suggesting their greater susceptibility to protonation and their role as primary anchoring sites for Au(iii) coordination.^29^ Electron localization function (ELF) analysis (Fig. 3c) showed distinct electronic behaviors: C–N sites exhibited localized electron density (low ELF values, blue isosurface), while C=N sites showed pronounced delocalization (high ELF values, red isosurface), confirming their function as electron-rich centers. Additionally, localized orbital locator (LOL) mapping highlighted the lone-pair electron characteristics of N atoms in C=N groups (Fig. S6). These findings confirm the effectiveness of C=N sites as Au(iii) recognition centers.

To investigate the dynamic electron transfer pathways, hole electron analysis was performed on the structural fragments.^30^ The Tapd units exhibited significant hole accumulation, while the imine-linked edges displayed electron-rich regions (Fig. 3d), clearly revealing a donor–acceptor–donor (D–A–D) architecture. Inter-fragment charge transfer (IFCT) analysis supported this mechanism (Table S2), showing that Tapd units contributed 0.552 e^−^, while imine bridges and Tpa accepted 0.261 e^−^ and 0.291 e^−^, respectively. These results confirm the D–A–D electronic configuration, which enhanced charge separation and directional electron transport.

Zeta potential analysis provides an intuitive reflection of the charge properties and charge density on the surface of the material. As depicted in Fig. 3e, the surfaces of all COFs display a positive charge at pH less than 5.0. Significantly, the 1D/2D hybrid materials play a role in optimizing the charge distribution on the material surface, thus further enhancing the positive-charge characteristics of the material. Consequently, Wurster-type COFs exhibited strong affinity for AuCl_4_^−^ and repulsion toward other metal cations, thereby efficiently suppressing co-ion repulsion and enhancing reduction selectivity.^31^

Electrochemical impedance spectra (EIS) indicate the more efficient charge separation ability and lower interfacial charge transfer resistance of TpaIda-2DCOF relative to other COFs (Fig. S7). The electron transfer capabilities of the four COFs were further investigated using UV-visible diffuse reflectance spectroscopy (UV-vis DRS). Compared to Tpa-2DCOF and Ida-1DCOF, TpaIda-2DCOF and IdaTpa-1DCOF exhibited a redshift in their absorption peak (Fig. 3f). Optical bandgap calculations (Fig. 3g) using the Tauc plot method revealed that TpaIda-2DCOF (1.70 eV) and IdaTpa-1DCOF (1.80 eV) had narrower bandgaps compared to Tpa-2DCOF (1.85 eV) and Ida-1DCOF (2.08 eV). This reduction in bandgap suggests that isomer incorporation facilitated charge transfer during redox processes. The narrower bandgap was attributed to the formation of a conjugated system within the Tapd-based D–A framework, which further enhanced electron transfer efficiency.^32^ Hall effect measurements confirmed that the four COFs are typical n-type semiconductors with negative Hall coefficients^33^ (Table S3). More importantly, TpaIda-2DCOF exhibited a Hall mobility of 1.59 cm^2^ V^−1^ s^−1^, which exceeded the 1.29 cm^2^ V^−1^ s^−1^ value observed for Tpa-2DCOF, further highlighting the improved charge transport capability of TpaIda-2DCOF (Fig. 3h), and is also 1 order of magnitude higher than that of previously reported n-type 2D COFs (Table S4). These results collectively demonstrate that regulating the local dimensional structure can effectively modulate charge transport properties in COFs.

The dimensional engineering strategy illustrated in Fig. 3i is likely to be the primary cause underlying the aforementioned alterations in the physical and chemical properties. Ida-1D COF forms corrugated nanofibrillar networks through staggered AB-stacking of molecular strips.^34^ While electron hopping occurred along the strip axis, charge transfer between chains was hindered by van der Waals gaps, limiting overall charge transfer efficiency. In contrast, crystalline sheets adopt an AA-stacked configuration in Tpa-2DCOF, where planar π-conjugated backbones engage in interlayer π–π stacking. This supramolecular arrangement enhances charge mobility between the interlayers.^35,36^ However, introducing linear monomers (*e.g.*, Tpa) into Ida-1DCOF facilitated covalent crosslinks between adjacent nanofibrils. These interstrand linkages established electron–exchange pathways, enhancing interchain charge transfer. Conversely, in Tpa-2DCOF, the introduction of nonlinear monomers (*e.g.*, Ida) disrupted AA stacking locally, forming pillar-supported bilayer structures. This topological reconfiguration creates through-pore charge transport channels, improving interlayer charge mobility.

### Reduction and recovery properties

**Fig. 4:**
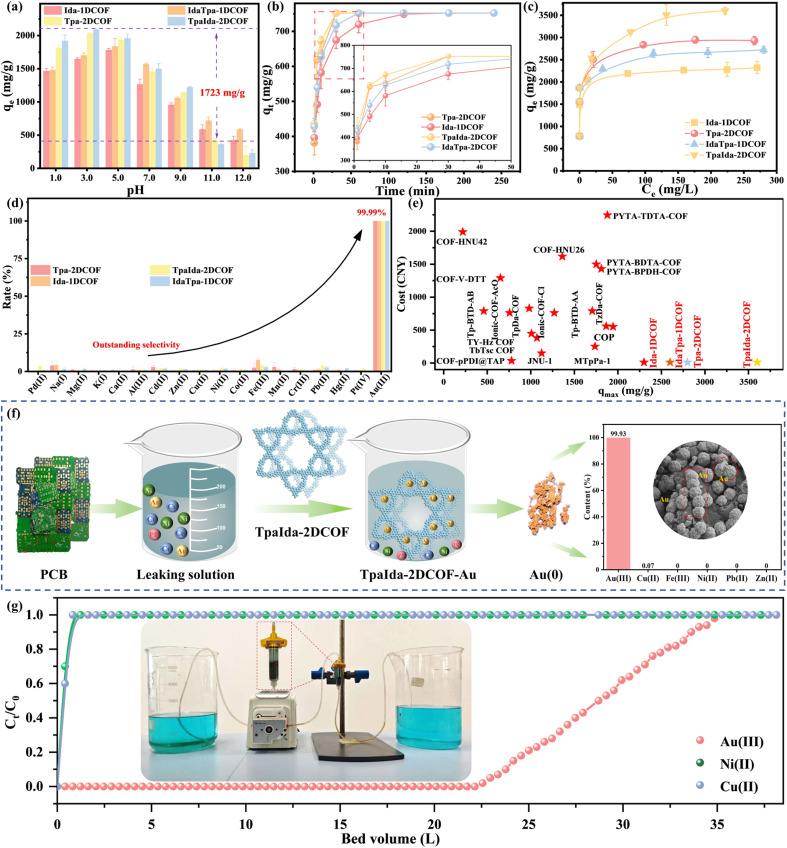
The recovery efficiency of Au(iii) under different pH conditions (a) and (b) time. (c) Au(iii) adsorption isotherms. (d) Au(iii) recovery efficiency of the four COFs in the presence of various interfering metal ions (50 ppm). Error bars represent the standard error of the mean. (e) Comparison of the maximum adsorption capacity and raw material cost with other COF-based adsorbents. (Reference information and abbreviations for comparative data are shown in Table S8.) (f) The experimental images of TpaIda-2DCOF for Au(iii) capture. Upon calcination at 850 °C, the resultant gold particles were dissolved in aqua regia, and the gold purity was determined to be 23.98 K. (g) The recovery of Au(iii) in the leaching solution by a fixed-bed experiment.

In view of the exceptional charge transfer efficiency expounded previously and the precisely tunable pore structure that can be meticulously designed, it is a potential candidate for Au(iii) recognition. To explore the factors influencing Au(iii) reduction and recovery efficiency, this study first examined the effect of pH on Au(iii) recovery. As shown in Fig. 4a, the capture ability was significantly influenced by pH, with a maximum difference of 1723 mg g^−1^ observed across different pH conditions. This behavior can be explained in two ways. First, the protonation of the material varied with pH, as confirmed by zeta potential measurements (Fig. 3e). Under acidic conditions, the imine groups were protonated, increasing the positive charge on the COF surface, which enhanced electrostatic interactions with the negatively charged Au(iii) species, such as AuCl_4_^−^. Second, the distribution of Au(iii) species changed with pH.^37^ In the low pH range (pH 1.0–4.0), Au(iii) primarily existed as the stable AuCl_4_^−^ species (Fig. S8). As pH increased, AuCl_4_^−^ gradually transformed into less stable species such as AuCl_3_OH^−^ and AuCl_2_OH_2_^−^, which were more easily reduced, thus contributing to an increase in the adsorption capacity. However, as the H^+^ concentration decreased significantly, particularly under alkaline conditions, the COF underwent deprotonation, which inhibited the redox reaction.

In addition, Fig. S9 shows that all recovery processes were endothermic, with increasing temperature significantly enhancing capture ability. Among the four COFs, Ida-1DCOF was the most sensitive to temperature changes, showing an increase of approximately 40 mg g^−1^ in capture ability for every 1 K increase. The correlation coefficient (*R*^2^) from the linear fitting was 0.999 (Fig. S10 and Table S5), indicating a strong linear relationship between temperature and capture ability. Additionally, a positive entropy change (Δ*S*^*θ*^ > 0) was observed, which can be attributed to the reduction of AuCl_4_^−^, releasing Cl^−^ into the solution and consequently increasing the disorder of the system.

To understand the kinetic behaviors of Wurster-type COFs, the adsorption curves were obtained (Fig. 4b). All COFs achieved complete Au(iii) recovery within 30 min, with adsorption capacities reaching 750 mg g^−1^. Kinetic analysis indicates that the adsorption process followed a pseudo-second-order model (Fig. S11–S14 and Table S6), suggesting that the process was mainly controlled by chemical reactions,^38,39^ likely involving the reduction of Au(iii). To confirm the reduction of Au(iii), the post-adsorption PXRD patterns were determined. Fig. S15 shows that the diffraction peaks corresponding to Au(0) dominated and matched the standard diffraction card (JCPDS no.04-0784), confirming the effective reduction of Au(iii) to elemental gold. Furthermore, SEM images of the adsorbent material reveal the presence of gold nanoparticles with diameters in the range of 60–120 nm on the surface (Fig. S16). These findings support the successful implementation of an *in situ* capture and recovery closed-loop process and suggest that redox-driven adsorption was the primary mechanism driving the Au(iii) capture.

The isothermal adsorption experiments (Fig. 4c) reveal that the maximum adsorption capacity of Ida-1DCOF was 2323 mg g^−1^, while Tpa-2DCOF exhibited a capacity of 2932 mg g^−1^. In contrast, the maximum adsorption capacities of IdaTpa-1DCOF and TpaIda-2DCOF reached 2720 mg g^−1^ and 3601 mg g^−1^. The enhanced adsorption performance was attributed to the improved electronic transport capabilities and optimized pore size and morphology, resulting from the local dimensional distinctions. This result shows that the synergistic effect of isomers can reconstruct the charge transfer pathway in COFs, improve the charge separation efficiency, and greatly enhance the mass transfer process of Au(iii). The fitting results (Fig. S17–S20 and Table S7) show a strong agreement with the Freundlich model (*R*^2^ = 0.998).

Ultrasonic-assisted exfoliation experiments were conducted to remove gold nanoparticles from the material surface and disperse the nanomaterials, exposing the active sites (Fig. S21). Stepwise measurements reveal that the maximum adsorption capacity reached 6900 mg g^−1^, which was significantly higher than the equilibrium adsorption capacity of 3601 mg g^−1^, indicating that ultrasonic assistance notably enhanced the adsorption capability (Fig. S22). Furthermore, acidic thiourea was used as an effective desorbing agent, achieving 99.9% desorption efficiency over twenty adsorption–desorption cycles (Fig. S23).

To comprehensively evaluate the selective recognition performance of Wurster-type COFs for Au(iii), selective recognition experiments were conducted using simulated solutions containing 18 metal ions, such as Au(iii), Pd(ii), Na(i), Mg(ii), K(i), Ca(ii), Al(iii), Cd(ii), Zn(ii), Cu(ii), Ni(ii), Co(ii), Fe(iii), Mn(ii), Cr(iii), Pb(ii), Hg(ii), and Pt(iv). Fig. 4d shows that the Au(iii) concentration in the solution dropped below the detection limit after adsorption, with the distribution coefficient reaching a maximum of 7.6 × 10^4^. In contrast, the removal rates for other metal ions were all below 5%, with distribution coefficients less than 3.5 (Fig. S24). Notably, even under the coexistence of Pd(ii) and Pt(iv), which have similar chemical properties to Au(iii), the selective recognition of Au(iii) remained unaffected. The adsorption capacities for Pd(ii) and Pt(iv) were below 10 mg g^−1^ (Fig. S25), and the Au(iii) selectivity coefficient was 2.8 × 10^6^ for Au(iii)/Pd(ii) and 1.1 × 10^7^ for Au(iii)/Pt(iv). Furthermore, Au(iii) was completely recovered under high ionic strength conditions (Fig. S26) or in the presence of multiple anions (Fig. S27). Notably, even in the presence of 500 mg L^−1^ Co(ii), Ni(ii), and Cu(ii), the Au(iii) recovery efficiency approached almost 100%, whereas the removal rates for these interfering metal ions remained consistently below 2% (Fig. S28 and S29).

Notably, the entire recycling process was demonstrated to be economically viable (Fig. 4e). TpaIda-2DCOF had a production cost of less than 10 CNY g^−1^, making it one of the most cost-efficient COF materials for gold recovery to date, with costs substantially lower than those of iIonic-COF-Br (762 CNY g^−1^) and PYTA-BDTA-COF (1499 CNY g^−1^). Additionally, with the current market price of gold (around 790 CNY g^−1^), combined with its exceptional gold extraction efficiency (over 3601 mg g^−1^), precise selectivity (*K*_d_ > 7.6 × 10^4^), simple synthesis process (one-pot, three-component synthesis strategy), and excellent chemical and thermal stability, (Fig. S30 and 31), TpaIda-2DCOF demonstrated strong economic potential. Although large-scale commercialization of these COFs remains in the developmental stage, their technical feasibility and economic viability for cost-effective gold recovery from electronic waste are clear.

Based on the excellent selectivity demonstrated above, we further conducted Au(iii) recognition experiments using real samples. The results show that just 4 mg of COF successfully reduced the Au(iii) concentration in leachates from a printed circuit board (PCB) (Fig. S32a), central processing unit (CPU) (Fig. S32b) and INFICON quartz chip (Fig. S32c) below the detection limit within 10 min, achieving nearly 100% recovery efficiency. Simultaneously, the removal rates for other elements remained below 2%. These findings highlight the outstanding selectivity recognition and interference resistance of Wurster-type COFs for Au(iii) recovery underscoring their potential for industrial applications.

Three main factors contribute to the excellent selectivity. First, the forms of metal ions in the solution differed significantly. Gold primarily existed as AuCl_4_^−^ complexes, while other base metals (*e.g.*, Cu, Fe, and Ni) were present as metal cations. This difference caused electrostatic repulsion between the positively charged COF material and the base metal cations, resulting in preferential adsorption of AuCl_4_^−^. Second, the standard electrode potentials (*E*^*θ*^) of different metal ions varied, as follows: standard redox potential for AuCl_4_^−^/Au^0^ (*E*^*θ*^ = 1.002 V) > PtCl_4_^−^/Pt^0^ (*E*^*θ*^ = 0.75 V) > PdCl_4_^−^/Pd^0^ (*E*^*θ*^ = 0.59 V) > Cu^2+^/Cu^0^ (*E*^*θ*^ = 0.34 V) > Fe^3+^/Fe^0^ (*E*^*θ*^ = −0.037 V) > Pb^2+^/Pb^0^ (*E*^*θ*^ = −0.126 V) > Ni^2+^/Ni^0^ (*E*^*θ*^ = −0.23 V) > Co^2+^/Co^0^ (*E*^*θ*^ = −0.277 V). Since AuCl_4_^−^/Au^0^ had the highest *E*^*θ*^ value, the COF material preferentially reduced AuCl_4_^−^, while other metal ions were adsorbed physically and could undergo redox reactions. These ions were displaced back into the solution due to the competitive nature of AuCl_4_^−^. Furthermore, no diffraction peaks assigned to other metals were observed in the PXRD spectra after adsorption, further confirming that these metals were not reduced (Fig. S33). Beyond electrostatic interactions and favorable redox potentials, the pore aperture size provides optimal molecular sieving effects for hydrated AuCl_4_^−^ ions while partially excluding larger hydrated metal complexes such as PtCl_6_^2−^. This size-exclusion effect, combined with the preferential electron transfer to Au(iii), explains the exceptional selectivity coefficients observed even in complex multi-metal solutions containing 18 competing ions.

To recover high-purity gold from discarded Printed Circuit Boards (PCBs), 100 PCBs were subjected to a leaching process. After adding 50 mg of TpaIda-2DCOF and stirring for 6 h, no Au was detected in the solution, indicating complete recovery of Au. The solid mixture was then calcined at 850 °C for 2 h and treated with hydrochloric acid. After dissolution in aqua regia, compositional analysis reveals the presence of 99.93% Au and 0.07% Cu. Despite the presence of Cu(ii) at a concentration of 50 g L^−1^ in the leachate, no other metal impurities were detected in the recovered gold (Fig. 4f). This was one of the highest purity gold achieved in such extraction processes to date. Such high purity directly translates to higher market value and eliminates the need for additional refining steps, substantially improving the overall economics of the recovery process. The fact that this purity was achieved from complex e-waste leachates containing numerous contaminants, rather than from synthetic single-metal solutions, underscores the remarkable selectivity of our dimensional engineering strategy and its potential to transform e-waste recycling economics. Moreover, the fixed-bed column packed with TpaIda-2DCOF as the packing material was able to completely process 35.2 L of low-concentration PCB leaching solution. The operating time extended up to 430 h (Fig. 4g), and the recovered gold exists in an elemental form. These data validate the successful fabrication of an integrated adsorption-enrichment and *in situ* reduction system, enabling a fully *in situ* capture and recovery closed-loop system.

### Mechanism exploration

**Fig. 5:**
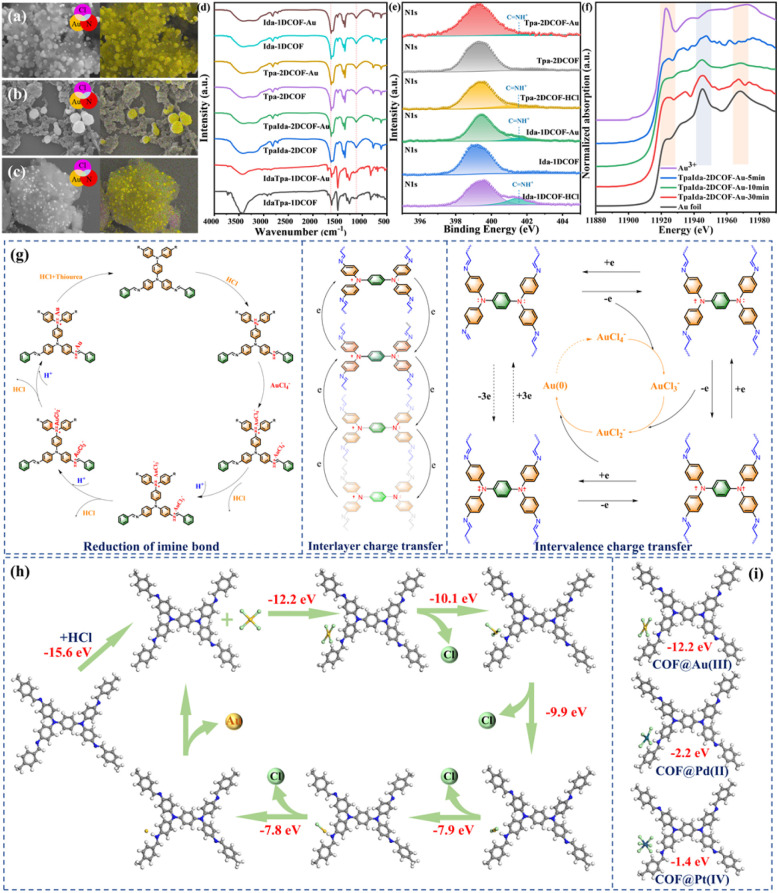
SEM images and elemental mapping of (a) TpaIda-2DCOF, (b) Ida-1DCOF, and (c) Tpa-2DCOF. (d) FT-IR spectra and (e) XPS N 1s spectra before and after adsorption. (f) The series of XANES spectra at the Au L3 edges of Au(iii) standard, Au foil, and samples with different contact times. (The spectra are shifted vertically for clarity.) (g) Schematic diagram illustrating the processes involving the reduction of Au(iii) *via* the imine bond, intervalence charge transfer, and the mechanism by which interlayer charge transfer contributes to the reduction of Au(iii). (h) The Au(iii) adsorption and reduction processes. (i) Binding energy of Pt(IV), Pd(ii), and Au(iii) adsorbed by the TpaIda-2DCOF unit.

After Au(iii) adsorption, energy dispersive X-ray (EDX) spectroscopy analysis revealed that the Au nanoparticles, with sizes ranging from 2 to 11 nm, were uniformly dispersed on the COF framework (Fig. 5a–c). The FT-IR spectra of Tpa-2DCOF and Tpa-2DCOF-Au showed no significant changes in the stretching vibrations of chemical bonds (Fig. 5d), indicating that the adsorption mechanism was primarily driven by the reduction of Au(iii), rather than electrostatic attraction or coordination interactions. The high-resolution XPS spectrum of N 1s demonstrated the appearance of a C=NH^+^ characteristic peak at 401.66 eV upon protonation (Fig. 5e). Notably, the intensity of this peak decreased significantly after adsorption, confirming the reduction of C=NH^+^ to C=N. In the high-resolution XPS spectrum of Au 4f, the peaks at 88.43 eV and 84.75 eV, corresponding to the 4f_5/2_ and 4f_7/2_ orbitals of Au(0), suggested the reduction of Au(iii) to Au(0). This reduction was likely facilitated by the protonated C=N bond, which promoted the conversion of Au(iii) to Au(0).^40,41^ The possible reduction pathway was proposed as follows: initially, protonated C=NH^+^ acted as active reduction sites, promoting the removal of Cl^−^ from AuCl_4_^−^ to form an AuCl_3_^−^ intermediate and release HCl. Subsequently, AuCl_3_^−^ bound with another C=NH^+^ group, reducing further to AuCl_2_^−^ and ultimately forming Au(0). Moreover, the Au L3-edge X-ray absorption near edge structure (XANES) results (Fig. 5f) reveal the complete stepwise reduction pathway from Au(iii) to Au(i) and finally to Au(0) in COF-based gold recovery systems, for the first time. This pathway is characterized by an increasing contact time, which results in the gradual weakening of the Au(iii) characteristic white line at 11 922 eV and the emergence of broad absorption edges at 11 948 eV and 11 970 eV, which are consistent with Au(0). Additionally, tertiary amine and unreacted terminal –NH_2_ groups may undergo similar chemical processes to facilitate reduction.

In addition to the well-established role of C=NH^+^ sites, we propose that electron transfer also plays a crucial role in Au(iii) reduction. The Wurster-type structure is highly prone to undergo single-electron oxidation, forming stable radical cations that act as charge carriers,^42,43^ thereby facilitating the reduction of Au(iii). Guo *et al.*^44^ previously discussed this oxidation–reduction process (Fig. S34). Specifically, the aromatic building units create a highly conjugated framework with π-electron-rich planes. When stimulated by external factors, stable charge carriers form within the D–A COF, promoting charge generation and transfer within the layers. Additionally, the stacked COF layers can form a vertical conductive system by adjusting the interlayer π–π overlap, thus enhancing charge transfer between layers. As a result, Au(iii) is reduced to Au(0), while the triphenylamine (TPA) undergoes oxidation to form radical cations. Electron paramagnetic resonance (EPR) spectroscopy confirms the presence of radical cations before and after the reaction (Fig. S35), providing further evidence for this proposed mechanism.

Fig. 5g depicts the process of Au(iii) reduction by intervalence charge transfer and interlayer charge transfer. Based on the above mechanisms, TpaIda-2DCOF exhibits superior mass transfer performance in Au(iii) reduction compared to Tpa-2DCOF due to its lower electrical resistivity and higher Hall mobility. From the perspective of structure–property relationships, this outstanding performance further confirms that the synergistic effects of isomers construct novel electron transport pathways, significantly enhancing the material's electron transport capability and ultimately facilitating the reduction and recovery of Au(iii).

pH solution experiments show that both of the above two reduction pathways play a role in acidic media. To investigate the critical role of H^+^ in the redox process, density functional theory (DFT) calculations were performed to determine the binding energies of H^+^ and AuCl_4_^−^ at two distinct active sites: tertiary amine nitrogen (site 1) and imine groups (site 2) (Fig. S36). Comparative calculations were also conducted for AuCl_4_^−^ adsorption following protonation. As depicted in Fig. S37, the binding energies for AuCl_4_^−^ adsorption without protonation were positive (2.33 eV for site 1 and 1.57 eV for site 2), whereas protonation prior to AuCl_4_^−^ adsorption resulted in significantly negative energy differences (−11.19 eV for site 1 and −12.24 eV for site 2). These results clearly indicate that AuCl_4_^−^ adsorption without protonation was energetically unfavorable, while protonation provided the necessary driving force for the reduction process.

The proposed reduction pathway of Au(iii) to Au(0) is illustrated in Fig. 5h. This process began with the protonation of C=N sites in acidic media, as supported by DFT calculations, which indicated an energy difference (Δ*E*) of 15.6 eV. The activated C=NH^+^ groups then reacted with AuCl_4_^−^, forming a C=N@AuCl_3_ intermediate through HCl elimination. Further protonation stabilized this intermediate as C=NH^+^@AuCl_3_. The stepwise removal of chloride ligands from the AuCl_3_ moiety facilitated the gradual reduction of Au(iii) to Au(0). This mechanism highlights the dual role of the COF framework: protonated C=N sites facilitated chloride elimination, while the conjugated structure promoted electron transfer, enabling efficient Au(iii) reduction under mild conditions. Additionally, as shown in Fig. 5i, the Pt(IV) and Pd(ii) adsorption at the same active site was energetically unfavorable, ensuring selective recovery of Au(iii).

## Experimental

### Materials and methods

Terephthalaldehyde (Tpa, ≥98.0%), isophthalaldehyde (Ida, ≥98.0%), tetrahydrofuran (THF, ≥99.5%), *N*,*N*,*N*′,*N*′-tetrakis(4-aminophenyl)-1,4-benzenediamine (Tapd, ≥97.0%), *n*-hexane (≥97.0%), sodium bromide (NaBr, ≥99.0%), 1-butanol (BuOH, 99.0%), 1,2-dichlorobenzene (*o*-DCB, ≥98.0%), ethanol (EtOH, 99.5%), sodium chloride (NaCl, 99.9%), sodium hydroxide (NaOH, ≥99.0%), and thiourea (99.0%) were acquired from Adamas-beta®, China. Hydrochloric acid (HCl, AR) was obtained from Chuandong Chemical Group, China. All reagents were used without additional purification.

### Synthetic procedure

A borosilicate glass tube was charged with Ida (13.4 mg, 0.10 mmol), Tapd (23.6 mg, 0.05 mmol), *o*-DCB (2.0 ml) and BuOH (2.0 ml) and was sonicated for 10 min. Aqueous AcOH (0.2 ml, 6.0 mol L^−1^) was then added to the mixture. Each tube was degassed through three freeze–pump–thaw cycles in a liquid nitrogen bath. The mixture was heated at 120 °C for 3 days. After cooling to room temperature, the precipitates were filtered and washed with THF, followed by further extraction in a Soxhlet extractor for 24 h using acetone, THF, and *n*-hexane. Among these solvents, *n*-hexane—selected for its low surface tension—was used to maintain the porosity and crystallinity.^45^ The solid was dried under vacuum at 80 °C overnight to yield Ida-1DCOF (95% yield). Meanwhile, the feed ratios of Tpa and Ida monomers were adjusted during synthesis to prepare TpaIda-2DCOF, TpaIda-2DCOF-3 : 1, TpaIda-2DCOF-2 : 1, IdaTpa-1DCOF, IdaTpa-1DCOF-3 : 1, and IdaTpa-1DCOF-2 : 1. Detailed synthesis procedures can be found in the SI.

## Conclusions

In summary, the directional transformation of the topological structure from 1D to 2D COFs was successfully achieved by developing a three-component synthesis strategy that regulated local dimensional interconnections. By precisely controlling the isomer ratio, the synergistic effect of isomers not only reconstructed the pore distribution and morphology of COFs but also enhanced interlayer charge transfer efficiency, significantly improving the electron transport kinetics of the COF network. Due to these structural improvements, the obtained 1D/2D heterostructure COFs exhibited an exceptionally high Au(iii) reduction and recovery capacity of 3601 mg g^−1^. Through a combination of experimental characterization studies and theoretical calculations, this study revealed that the protonated C=N bond served as the key active site for Au(iii) reduction, and clarified the role of triphenylamine groups in promoting the reduction reaction through single-electron transfer. Finally, synchrotron radiation characterization confirmed the stepwise reduction pathway of gold species (Au(iii) → Au(i) →Au(0)). This work not only highlighted the unique advantages of Wurster-type COFs in precious metal recovery but also provided a new method for optimizing the topological structure and charge distribution of COFs through isomer ratio regulation, verifying the feasibility of the host–guest recognition system for an *in situ* capture and recovery closed-loop process for Au(iii).

## Author contributions

Jiaxing Xiong: investigation, formal analysis, software, writing – original draft. Qi An: writing – review & editing. Hao Xiang: conceptualization. Yu Zhou: formal analysis. Yuan Zhang: investigation. Wenjing Chen: formal analysis. Boxian Ren: methodology. Shixiong Wang: supervision. Huiping Bai: funding acquisition. Hong Guo: writing – review & editing. Xiangjun Yang: funding acquisition, writing – review & editing.

## Conflicts of interest

There are no conflicts to declare.

## Supplementary Material

SC-OLF-D5SC05206H-s001

## Data Availability

Additional data are available from the corresponding author upon request. The data that support the findings of this study are available within the paper and its supplementary information (SI). Supplementary information: experimental details, characterization data, and computational information. See DOI: https://doi.org/10.1039/d5sc05206h.
