# Supplementary material for: Topology engineering of COFs via localized 1D–2D unit interconnection to facilitate interfacial electron transfer for efficient gold recovery from e-waste leachates
Source: Chem Sci. 2025 Nov 7;17(1):436–47. doi: 10.1039/d5sc05206h (PMC12616540; doi:10.1039/d5sc05206h)
Supplement: SC-017-D5SC05206H-s001 [file SC-017-D5SC05206H-s001.pdf]

Supplementary Information (SI) for Chemical Science.
This journal is © The Royal Society of Chemistry 2025

Topology Engineering of COFs via Localized 1D-2D Unit Interconnection to Facilitate Interfacial Electron Transfer for Efficient Gold Recovery from E-Waste Leachate

Jiaxing Xiong[a], Qi An[b], Hao Xiang[a], Yu Zhou[a], Yuan Zhang[a], Wenjing Chen[a], Boxian Ren[a], Shixiong Wang[a,], Huiping Bai[b,]*, Hong Guo[b,]*, Xiangjun Yang[a,]*.

[a]School of Chemical Science and Technology, Yunnan University, Kunming, 650091, China.

[b]International Joint Research Center for Advanced Energy Materials of Yunnan Province, School of Materials and Energy, Yunnan University, Kunming 650091, China.

* Corresponding Authors: E-mail: baihuiping@ynu.edu.cn; guohong@ynu.edu.cn; yxjun@ynu.edu.cn;

Content

Figure S1. The PXRD patten of TpaIda-COP. 11
Figure S2. The PXRD patten of COFs formed by the reaction of Tpa, Ida and Tapd in different proportions 11
Figure S3. The PXRD patten of Tpa-2DCOF and TpaIda-2DCOF with the 2 Theta range of 1.0-10.0 (degree) 12
Figure S4. XPS spectra of N 1s for TpaIda-1DCOF, Ida-1DCOF and Tpa-2DCOF 12
Figure S5. The SEM of TpaIda-2DCOF-3:1, TpaIda-POP and IdaTpa-1DCOF-3:1 12
Figure S6. Localized orbital locator mapping for unit of TpaIda-2DCOF. 13
Figure S7. Electrochemical impedance spectra of Ida-1DCOF, Tpa-2DCOF IdaTpa-1DCOF and TpaIda-2DCOF 13
Figure S8. The variation of Au(III) species with pH levels. 14
Figure S9. The recovery efficiency of Au(III) under different temperatures. 14
Figure S10. Van der Hoff plots for Au(III) adsorption of Ida-1DCOF, Tpa-2DCOF IdaTpa-1DCOF and TpaIda-2DCOF. Error bars represent the standard error of the mean. 15
Figure S11. The fitting results of Pseudo-first-order model and pseudo-second-order model for IdaTpa-1DCOF 15
Figure S12. The fitting results of Pseudo-first-order model and pseudo-second-order model for Ida-1DCOF 16
Figure S13. The fitting results of Pseudo-first-order model and pseudo-second-order model for Tpa-2DCOF 16
Figure S14. The fitting results of Pseudo-first-order model and pseudo-second-order model for TpaIda-2DCOF 17
Figure S15. PXRD pattern before and after adsorption. 17
Figure S16. The SEM imaging of Tpa-2DCOF, TpaIda-2DCOF, IdaTpa-1DCOF and Ida-1DCOF after adsorption. 18
Figure S17. The fitting results of Langmuir model and Freundlich model for Ida-1DCOF. 18
Figure S18. The fitting results of Langmuir model and Freundlich model for IdaTpa-1DCOF. 19
Figure S19. The fitting results of Langmuir model and Freundlich model for TpaIda-2DCOF. 19
Figure S20. The fitting results of Langmuir model and Freundlich model for Tda-2DCOF. 20
Figure S21. The SEM of Tpa-2DCOF(a), Tpa-2DCOF-Au before (b) and after(c) sonication. 20
Figure S22. Ultrasound-assisted peeling experiment. Error bars represent the standard error of the mean. 20
Figure S23. The Au(III) removal efficiency of Tpa-2DCOF for 20 cycles. Error bars represent the standard error of the mean. 21
Figure S24. The distribution coefficients of different ions. Error bars represent the standard error of the mean. 21
Figure S25. Effect of Pt(IV) and Pd(II) on Au(III) adsorption. Error bars represent the standard error of the mean. 22
Figure S26. Effect of ionic strength on Au(III) adsorption. Error bars represent the standard error of the mean. 22
Figure S27. Effects of different anions on Au(III) adsorption. Error bars represent the standard error of the mean. 23

Figure S28. Effect of different concentrations of Cu(II), Ni(II) and Zn(II) on Au(III) adsorption. Error bars represent the standard error of the mean. 23
Figure S29. Removal of Cu(II), Ni(II) and Zn(II) at different concentrations. Error bars represent the standard error of the mean. 24
Figure S30. PXRD patterns of IdaTpa-1DCOF before and after treatment under DMF, 0.1 M HCl and 0.1 M KOH for 1 days. 24
Figure S31. TGA curve of the Tpa-2DCOF, Ida-1DCOF, IdaTpa-1DCOF and TpaIda-2DCOF. 25
Figure S32. Adsorption performance of four COFs in (a) PCB leaching solution, (b) CPU leaching solution and (c) INFICON quartz chips. 26
Figure S33. PXRD patterns of different COFs after the adsorption process. 27
Figure S34. Schematic illustration of the proposed oxidation/reduction pathway of Tpa-2DCOF (IVCT: intervalence charge transfer, ILCT: interlayer charge transfer). 27
Figure S35. Electron paramagnetic resonance spectroscopy of TpaIda - 2DCOF. (Blue line) Before adsorption; (Red line) After adsorption. 28
Figure S36. Distribution diagram of active sites. 28
Figure S37. The reaction coordinate for different adsorption sites. 29
Figure S38. FT - IR spectra of IdaTpa - 1DCOF during Au(III) adsorption at different times. 29
Figure S39. Top view and side-view projected along its c and b axis of the crystal lattices for Ida-1DCOF with sql-staggered stacking models. 30
Figure S40. Top view and side-view projected along its c and b axis of the crystal lattices for Ida-1DCOF with sql-parallel stacking modes. 30
Figure S41. Top view and side-view projected along its c and b axis of the crystal lattices for Tpa-2DCOF with kgm-AA stacking models. 31
Figure S42. Top view and side-view projected along its c and b axis of the crystal lattices for Tpa-2DCOF with kgm-AB stacking models. 31
Figure S43. Top view and side-view projected along its c and b axis of the crystal lattices for TpaIda-2DCOF with kgm-AA stacking models. 32
Figure S44. Top view and side-view projected along its c and b axis of the crystal lattices for TpaIda-2DCOF with kgm-AB stacking models. 32
Table S1. Elemental analysis. 33
Table S2. Transferred electrons between fragments. 33
Table S3. Hall effect. 33
Table S4. Summary of the intrinsic charge mobilities of COF materials at ambient temperature reported in literature. 34
Table S5. Thermodynamic parameters. 34
Table S6. Kinetic parameters. 34
Table S7. Isotherm parameters. 35
Table S8. Comparison of adsorption capacity and cost of different materials. 36

1． Experimental Procedures

1.1 COF synthesis

Synthesis of Ida-1DCOF

A borosilicate glass tube measuring 10 × 12 mm (i.d. × o.d.) was charged with Ida (13.4 mg, 0.10 mmol), Tapd (23.6 mg, 0.05 mmol), o-DCB (2.0 ml) and BuOH (2.0 ml) and sonicated for 10 min. After that, aqueous AcOH (0.2 mL, 6.0 mol/L) was added to the mixture. Each tube was degassed by three freeze-pump-thaw cycles in a liquid nitrogen bath. The length of the tube was reduced to around 8 cm on sealing. After warming to room temperature, the mixture was heated at 120 °C for 3 days in an oven to yield a yellow solid. After cooling to room temperature, the yellow precipitates were filtered and washed with THF, then the yellow solid described above was transferred into a tea bag and further washed with acetone, THF and n-Hexane for 24 h in a Soxhlet extractor. The solid was dried under vacuum at 80 °C overnight to yield Ida-1DCOF (95% yield).

Synthesis of Tpa-2DCOF

A borosilicate glass tube measuring 10 × 12 mm (i.d. × o.d.) was charged with Tpa (13.4 mg, 0.10 mmol), Tapd (23.6 mg, 0.05 mmol), o-DCB (2.0 ml) and BuOH (2.0 ml) and sonicated for 10 min. After that, aqueous AcOH (0.2 mL, 6.0 mol/L) was added to the mixture. Each tube was degassed by three freeze-pump-thaw cycles in a liquid nitrogen bath. The length of the tube was reduced to around 8 cm on sealing. After warming to room temperature, the mixture was heated at 120 °C for 3 days. After cooling to room temperature, the precipitates were filtered and washed with THF, then the solid described above was transferred into a tea bag and further washed with acetone, THF and n-Hexane for 24 h in a Soxhlet extractor. The solid was dried under vacuum at 80 °C overnight to yield Tpa-2DCOF (93% yield).

Synthesis of TpaIda-COP

A borosilicate glass tube measuring 10 × 12 mm (i.d. × o.d.) was charged with Ida (6.7 mg, 0.05 mmol), Tpa (6.7 mg, 0.05 mmol), Tapd (23.6 mg, 0.05 mmol), o-DCB (2.0 ml) and BuOH (2.0 ml) and sonicated for 10 min. After that, aqueous AcOH (0.2 mL, 6.0 mol/L) was added to the mixture. Each tube was degassed by three freeze-pump-thaw cycles in a liquid nitrogen bath. The length of the tube was reduced to around 8 cm on sealing. After warming to room temperature, the mixture was heated at 120 °C for 3 days. After cooling to room temperature, the precipitates were filtered and washed with THF, then the solid described above was transferred into a tea bag and further washed with acetone, THF and n-Hexane for 24 h in a Soxhlet extractor. The solid was dried

under vacuum at 80 °C overnight to yield TpaIda-COP (93% yield) .

Synthesis of TpaIda-2DCOF-n

This synthesis only changed the ratio of Tpa to Ida monomers, and other synthesis conditions were consistent with the synthesis conditions of Tpa-2DCOF. Synthesized with Ida (2.7 mg, 0.02 mmol), Tpa (10.7 mg, 0.08 mmol) and Tapd (23.6 mg, 0.05 mmol) named TpaIda-2DCOF. Synthesized with Ida (3.4 mg, 0.025 mmol), Tpa (10.0 mg, 0.075 mmol) and Tpda (23.6 mg, 0.05 mmol) named TpaIda-2DCOF-3:1. Synthesized with Ida (4.4 mg, 0.033 mmol), Tpa (9.0 mg, 0.067 mmol) and Tpda (23.6 mg, 0.05 mmol) named TpaIda-2DCOF-2:1.

Synthesis of IdaTpa-1DCOF-n

This synthesis only changed the ratio of Tpa to Ida monomers, and other synthesis conditions were consistent with the synthesis conditions of Tpa-2DCOF. Synthesized with Tpa (2.7 mg, 0.02 mmol), Ida (10.7 mg, 0.08 mmol) and Tapd (23.6 mg, 0.05 mmol) named IdaTpa-1DCOF. Synthesized with Tpa (3.4 mg, 0.025 mmol), Ida (10.0 mg, 0.075 mmol) and Tpda (23.6 mg, 0.05 mmol) named IdaTpa-1DCOF-3:1. Synthesized with Tpa (4.4 mg, 0.033 mmol), Ida (9.0 mg, 0.067 mmol) and Tpda (23.6 mg, 0.05 mmol) named IdaTpa-1DCOF-2:1.

1.2 Structural and chemical characterization

The morphologies and structures of the samples were characterized by using field emission scanning electron microscopy (SEM, Nova Nano SEM 450, FEI, USA) with an energy dispersive X-ray spectrometer (EDS) and the transmission electron microscopy (TEM, JEM-2100, JEOL Co., Japan) operated at an accelerating voltage of 100 kV. X-ray photoelectron spectroscopy (XPS) was performed on Thermo Scientific ESCALAB 250Xi with AlKα radiation. Fourier transform infrared (FT-IR) data were performed on a Nicolet iS10 spectrometer (Thermo Fisher Scientific Co). C, N, O content was quantified using an elemental analyzer (EA, Flash EA 2000 CHNS, Thermo Scientific, USA).Thermogravimetric analysis (TGA) was recorded on a (DSC/1600LF, ETTLER TOLEDO, Swiss Confederation) thermal analyzer under N_2. The operational range of the instrument was from 30 °C to 800 °C at a heating rate of 10 °C $min^{-1}$with an N_2 flow rate of 30 mL min^{-1}. The phase composition and crystal structures were assessed via powder X-ray diffraction (PXRD) using CuKα radiation (D8 Venture, Bruker, Germany) over the range of 2θ = 2-40° with a step size of 5°/min. Zeta potentials were measured on a Zeta sizer (Malvern). Metal ion concentrations were determined using an inductively coupled plasma optical emission spectrometer (ICP-OES-Optima 8300,

PerkinElmer, USA).

1.3 **Computational details**

The geometry optimization and density functional theory (DFT) chemical description for the molecular structures of all compounds were performed using Gaussian 16 package[1] with tight SCF convergence and ultrafine integration grids. The DFT method was employed with the SDD pseudopotential basis set for Pd, Pt ,Au and 6-311G(d, p) basis set for other atoms. The LUMO, HOMO and ESP of the unit were calculated. The Multiwfn 3.8[2] program developed by Lu et al[3] was used for wavefunction analysis. The isosurface maps were generated using VMD 1.9.3 software[4]. As such, four different clusters are considered: four ditopic building units corresponding to the tetratopic building units corresponding to the linear linker or V-shaped linear and N, N, N', N'-Tetrakis(4-aminophenyl)-1,4-benzenediamine linker, each with a suitable termination to mimic the environment of the moiety in the periodic framework. Because of the use of a simplified model, this may lead to the underestimation of long-range electronic delocalization effects or interfacial electric field effects.

The possible structure simulation of four COFs was carried out in Accelrys Materials Studio software package. The resulting structure was geometrically optimized using the Forcite module, with a universal forcefield and charge using Q_{eq}, respectively. The simulated XRD patterns were determined by the Reflex module. Pawley refinement was conducted to optimize the lattice parameters iteratively until the R_{wp} value converge

The adsorption mechanism of $AuCl_4^-$ on the TpaIda-2DCOF surface was investigated based on the Dmol 3 module of the Material Studio software package. The Generalized gradient approximation (GGA) method with the Perdew-Burke-Ernzerhof (PBE) was adopted as the exchange correlation functional. The convergence criteria for the plan optimization are: change in system energy less than 1×10^{-5} Ha; change in system forces less than 0.002 Ha/Å; and change in displacements less than 5×10^{-3}Å. The convergence accuracy of the self-consistent iteration is such that the energy change is less than 1×10^{-6}. Also, the truncation radius of the system calculations is set to 5.0 Å to take into account the accuracy of the calculations and the computational cost. The total interaction energy (Eint-1) of Tpa-2DCOF with $AuCl_4^-$ was estimated using the following relationship:

$$\text{Eint-1} = E(\text{COF-unit - }AuCl_4^-) - E(\text{COF-unit}) - E(AuCl_4^-) \quad (1)$$

where E(COF-unit), ($AuCl_4^-$), and E(COF-unit-$AuCl_4^-$) are the total energies of a given COF-unit, an isolated $AuCl_4^-$ anion and adsorbed on the given COF-unit, respectively.

1.4 Adsorption experiments

Preparation of gold stock solution

The gold stock solution is prepared by dissolving gold powder in freshly prepared aqua regia, a 1:3 molar mixture of concentrated nitric acid and hydrochloric acid. The gold powder is accurately weighed, transferred to a beaker, and dissolved under gentle stirring within a fume hood to manage the release of gases. Following complete dissolution, the solution is purified by adding a 1:1 hydrochloric acid-water mixture, which is heated to near dryness to remove NO_3^-. This cycle is repeated several times. Finally, the solution is diluted with ultrapure water to the desired concentration and stored in a tightly sealed container for further use. Lower concentrations were made through serial dilutions of the stock solution.

Adsorption isotherms

Different concentrations of Au(III) aqueous solutions were prepared using stock solution. 2 mg of adsorbent was mixed with 20 ml Au(III) solutions of different concentrations of Au(III) solution (50-500 ppm). The solutions were then stirred overnight to achieve equilibrium. The solutions were filtered through a 0.22 um membrane filter and the filtrate was analyzed via ICP-OES to determine the residual Au concentrations. The amount adsorbed or uptake capacity, q_e (mg g^{-1}), at equilibrium was calculated using equation 2.

$$q_e=\frac{(C_0-C_e)V}{m} \quad (2)$$

$$R=\frac{(C_0-C_e)}{C_0}\times 100\% \quad (3)$$

where q_e is the amount of adsorbed Au(III) (mg g^{-1}). C_0 (mg L^{-1}) and C_e (mg L^{-1}) are the initial and equilibrium concentrations of the metal ions, respectively. V (L) refers to the volume of the solution and m (g) is the mass of the adsorbent.

The Langmuir isotherm is based on the assumption that the adsorbent can only be adsorbed in a single layer on the adsorbent. The linear fitting of the Langmuir isotherm model is represented as following equation (4):

$$q_e=\frac{q_{max}\times C_e\times K_L}{1+C_e\times K_L} \quad (4)$$

where q is the maximum adsorption when the adsorption reaches equilibrium, and K_L(L mg^{-1}) is a constant characterized by the affinity of the adsorbate with the adsorbent. The value of C_e/q_e as the function of C_e were plotted and fitted with a linear equation from which the q_m and k_L. could be calculated according to the slope and intercept.

The Freundlich model is an empirical equation based on adsorption on a heterogeneous surface. The linear fitting of the Freundlich isotherm model is expressed by equation 5:

$$q_e = K_F C_e^{1/n} \quad (5)$$

where K_F denotes the Freundlich sorption coefficient, and n expresses how favorable the sorption process is K_F and n are empirical coefficients.

Adsorption kinetics

2 mg of COF were added to centrifuge tube containing 20 mL of a 100 ppm Au(III) solution. At increasing time intervals of 2 mL, aliquots were removed from the mixtures filtered through a 0.22 um membrane filter, and ICP-OES analyzed the filtrates for the remaining Au(III) concentration. The adsorption capacity at time (q_t, mg g^{-1}) is calculated by formula (2). The removal efficiency (Re, %) is calculated as the following formula (3).

Pseudo-first-order kinetic model is described as the following equation (6).

$$\ln(q_e - q_t) = \ln q_e - k_F t \quad (6)$$

where q_e represents the amount of Au(III) on the adsorbent under equilibrium, and k_F (min^{-1}) is pseudo-first-order adsorption rate constant.

Pseudo-second-order kinetic model is described as the following equation (7):

$$\frac{t}{q_t} = \frac{1}{k_s \times q_e^2} + \frac{t}{q_e} \quad (7)$$

where q_e represents the amount of Au(III) on the adsorbent under equilibrium, and k_s, (g mg^{-1} min^{-1}) is pseudo-second-order adsorption rate constant.

Adsorption thermodynamics

The thermodynamic parameters, such as standard entropy change (ΔS^0), standard enthalpy change (ΔH^0), standard Gibbs free energy (ΔG^0), and thermodynamic equilibrium constant (K), were determined using Van't Hoff equation, as shown in equations (8)–(10)[5].

$$K=\frac{q_e}{c_e} \tag{8}$$

$$\ln K=-\frac{\Delta H^{\theta}}{RT}+\frac{\Delta S^{\theta}}{R} \tag{9}$$

$$\Delta G^{\theta}=\Delta H^{\theta}-T\Delta S^{\theta} \tag{10}$$

Selectivity in multi-element solutions

2 mg of COF were added to centrifuge tube containing 20 mL of a mixed metal solution containing 50 ppm Au(III), Fe(III), Zn(II), Pb(II), Ni(II), Pd(II), Pt(IV), Na(I), Mg(II),Ca(II), Cu(II), Mn(II), Cr(III), Hg(II), K(I), Co(II), Al(III) and Cd(III). The solutions were stirred overnight to achieve equilibrium. The solutions were filtered through a 0.22 um membrane filter and the filtrate was analyzed via ICP-OES to determine the residua Au(III) concentrations. The distribution coefficients (K_d) after overnight treatment were calculated using equations 11-12.

$$K_d=\frac{(C_0\text{-}C_e)}{c_e}\frac{v}{m} \tag{11}$$

$$\beta=\frac{K_{d(Au)}}{K_{d(coexisting\ ions)}} \tag{12}$$

COF regeneration

Au-contacted adsorbents were placed in 20 mL of a 5% thiourea and 0.1M HCl solution and stirred overnight. The COF was then filtered and rinsed with water. The adsorbents were filtered washed with water, and dried under vacuum.

pH

The adsorbents were added to 20 mL of 100 ppm Au(III) solutions of pH 1.0 – 11.0. The solutions were stirred overnight to achieve equilibrium. The solutions were filtered through a 0.22 um membrane filter and the filtrate was analyzed via ICP-OES to determine the residual Au(III) concentrations.

Practical application of gold recovery from e-waste treated with aqua regia.

100 PCBs were soaked in 200 mL of aqua regia for 24 h. The leaching solution was obtained by filtration and diluted with water to a volume of 500 mL. After that, the pH of the leachate was changed to 2.0 by adding NaOH. TpaIda-2DCOF was soaked in a highly concentrated simulated electronic waste leaching solution until equilibrated and the resulting powder underwent PXRD to confirm the presence of neutral state Au. The mother liquor was filtered using a syringe filter with 0.22 μm pores to remove any solids for subsequent elemental analysis. The resulting composite/Au

powder was then loaded in a tube furnace and heated to 900 °C at a ramp of 30 °C per minute in air. The temperature was held for 2 hours and then allowed to cool to room temperature. The brown powder was transferred to a vial and 10 mL of concentrated HCl was added. The Au particles were then separated from the clear acidic solution and washed with distilled water 3 times. The purified Au was then dissolved in aqua regia and the purity of gold was determined by ICP-OES.

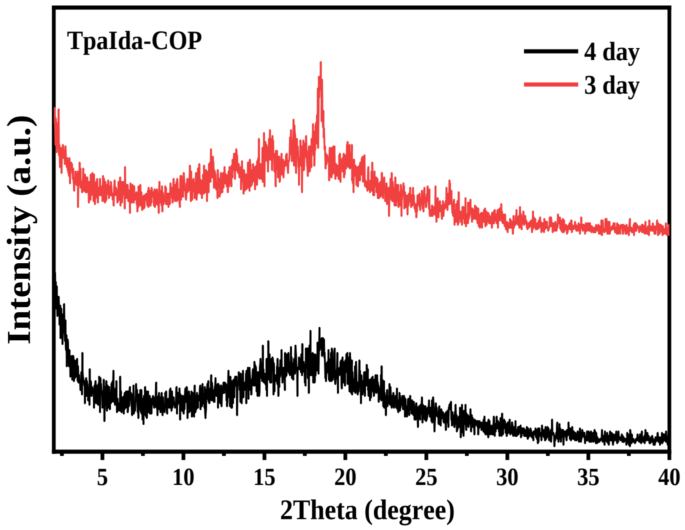

Figure S1. The PXRD patten of TpaIda-COP.

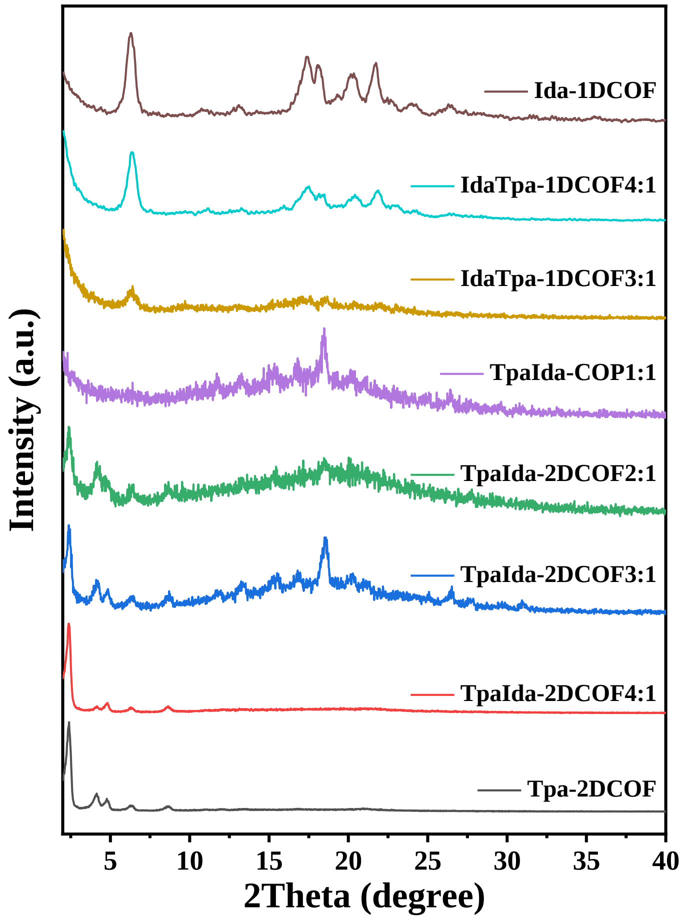

Figure S2. The PXRD patten of COFs formed by the reaction of Tpa, Ida and Tapd in different proportions.

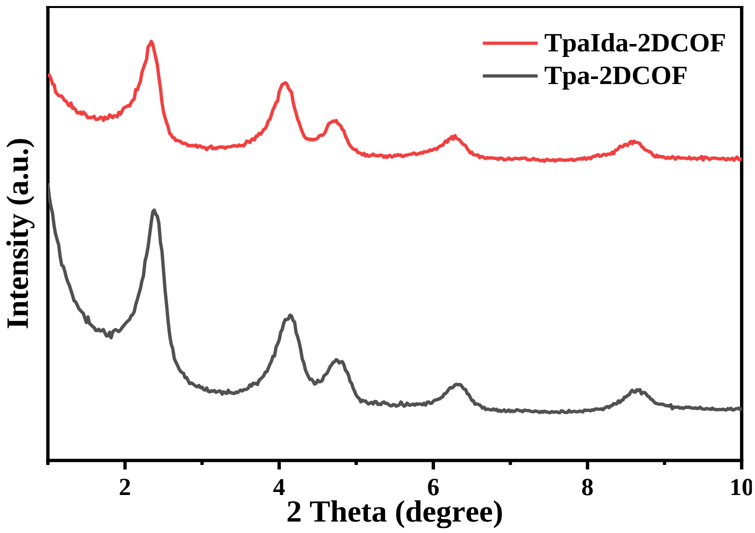

Figure S3. The PXRD patten of Tpa-2DCOF and TpaIda-2DCOF with the 2 Theta range of 1.0-10.0 (degree).

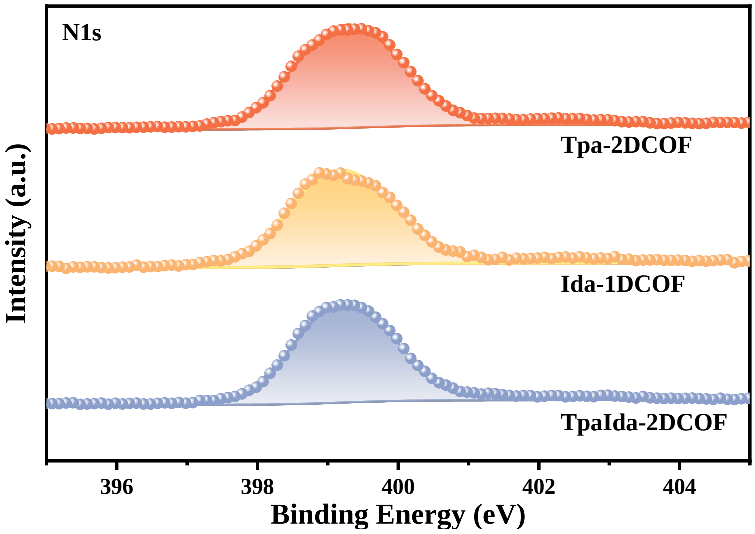

Figure S4. XPS spectra of N 1s for TpaIda-1DCOF, Ida-1DCOF and Tpa-2DCOF.

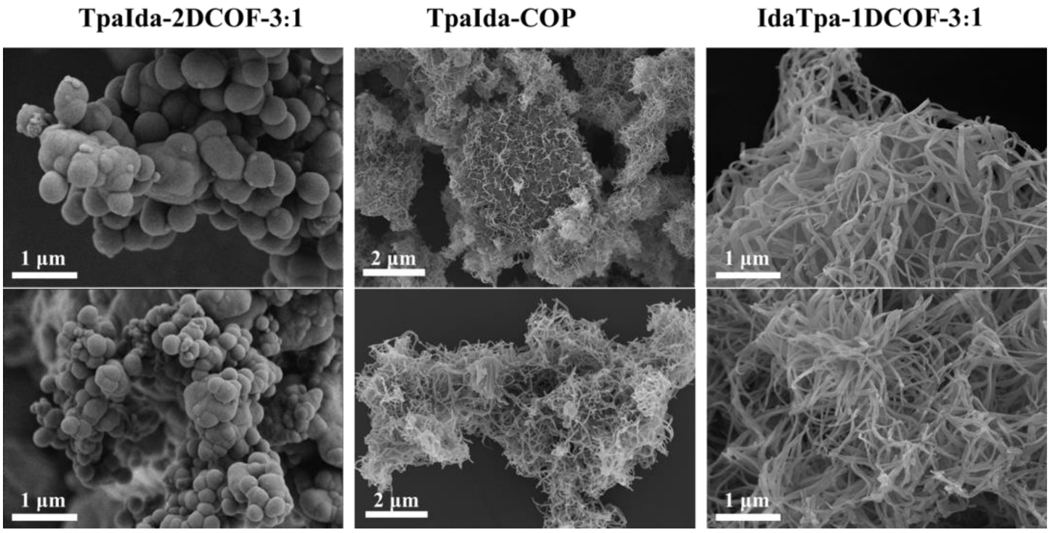

Figure S5. The SEM of TpaIda-2DCOF-3:1, TpaIda-POP and IdaTpa-1DCOF-3:1.

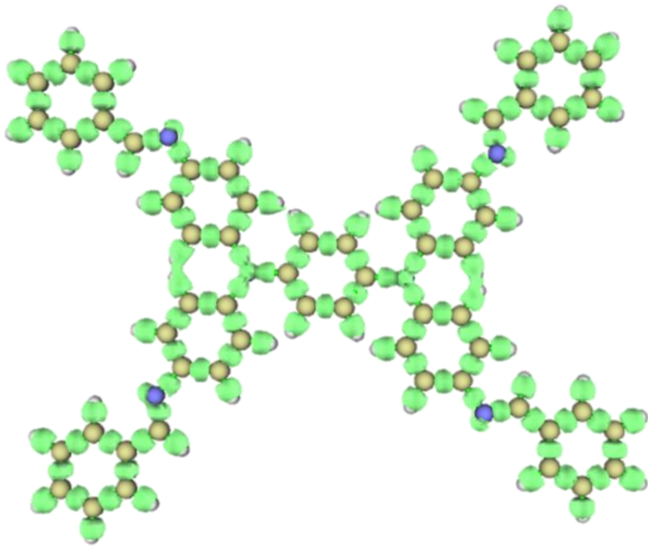

Figure S6. Localized orbital locator mapping for unit of TpaIda-2DCOF.

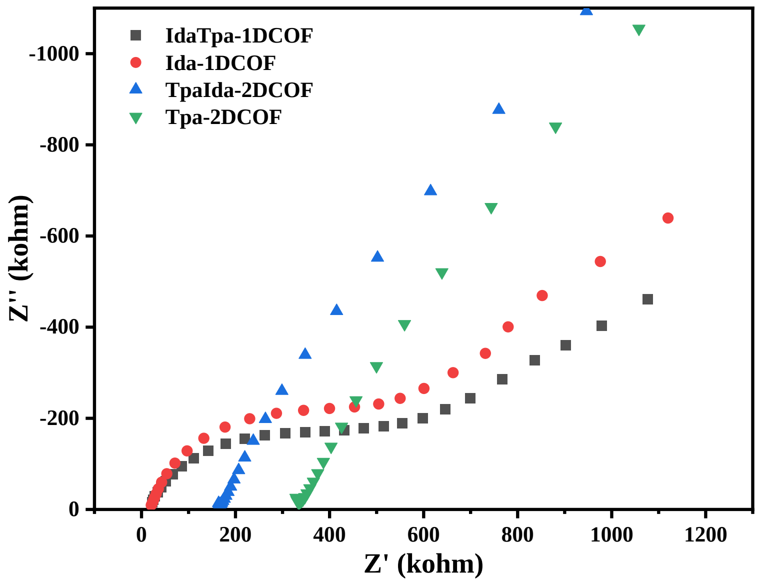

Figure S7. Electrochemical impedance spectra of Ida-1DCOF, Tpa-2DCOF IdaTpa-1DCOF and TpaIda-2DCOF.

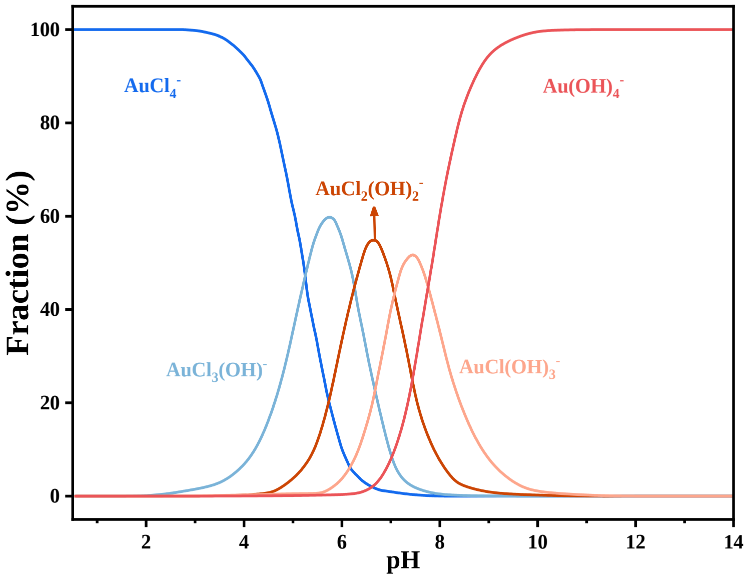

Figure S8. The variation of Au(III) species with pH levels.

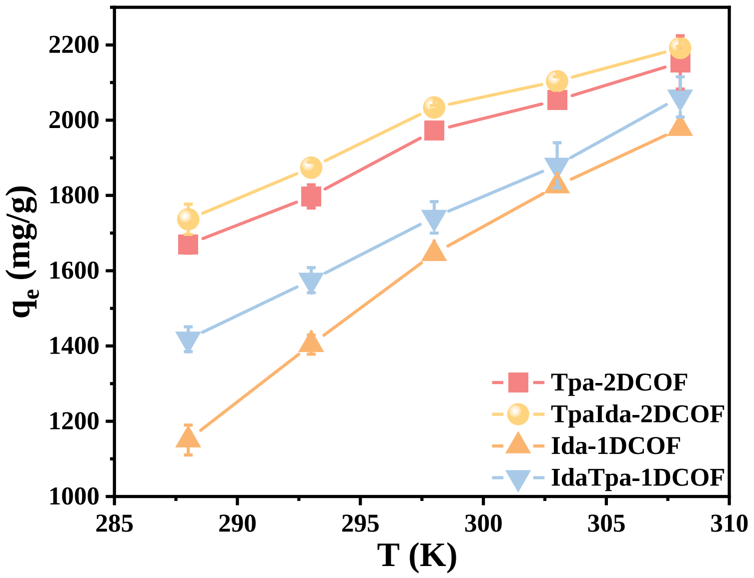

Figure S9. The recovery efficiency of Au(III) under different temperatures.

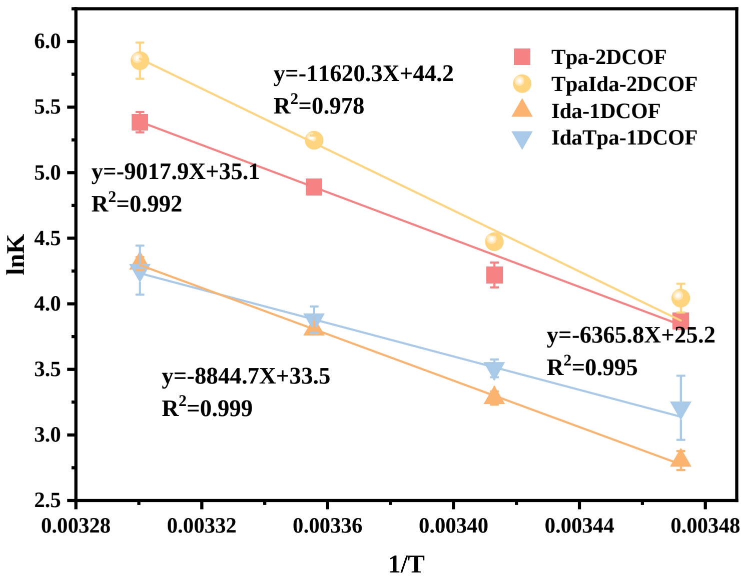

Figure S10. Van der Hoff plots for Au(III) adsorption of Ida-1DCOF, Tpa-2DCOF IdaTpa-1DCOF and TpaIda-2DCOF. Error bars represent the standard error of the mean.

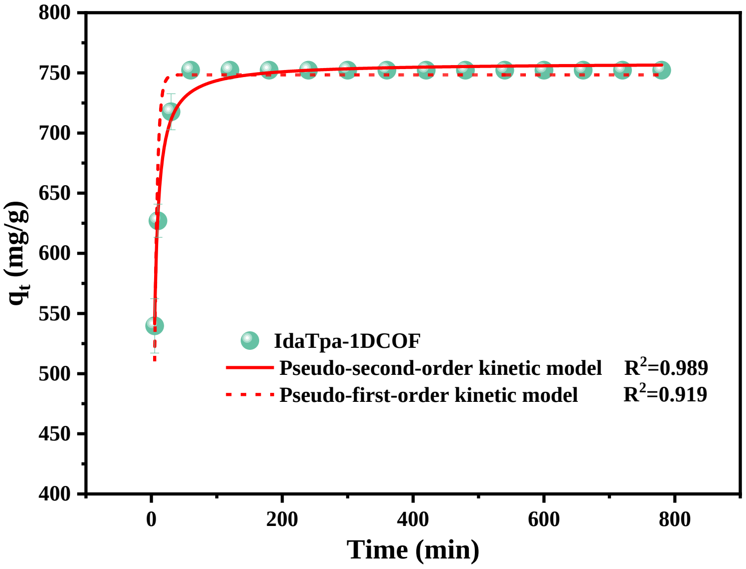

Figure S11. The fitting results of Pseudo-first-order model and pseudo-second-order model for IdaTpa-1DCOF.

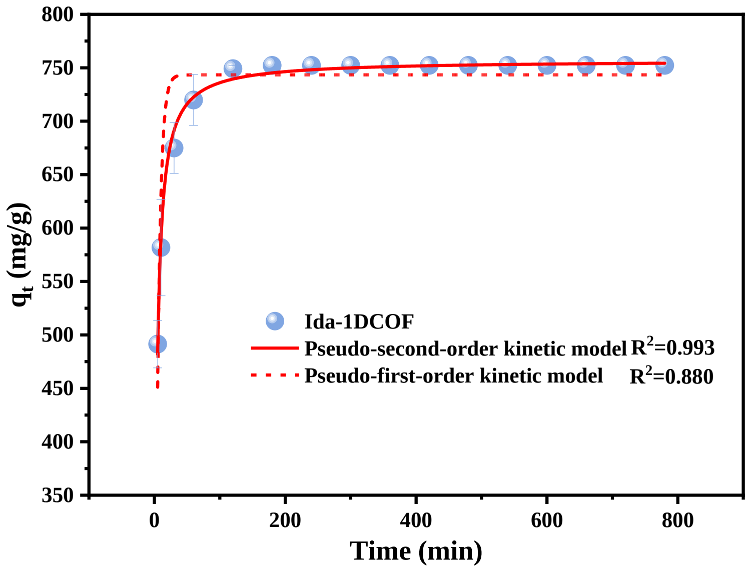

Figure S12. The fitting results of Pseudo-first-order model and pseudo-second-order model for Ida-1DCOF.

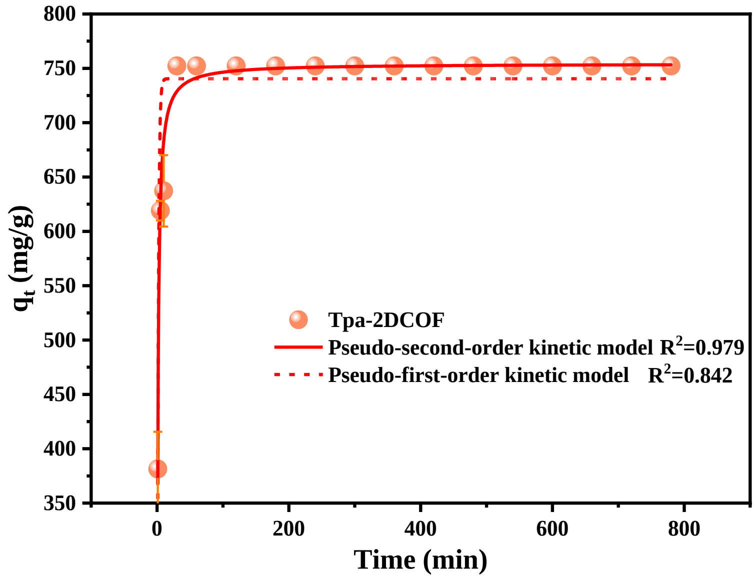

Figure S13. The fitting results of Pseudo-first-order model and pseudo-second-order model for Tpa-2DCOF.

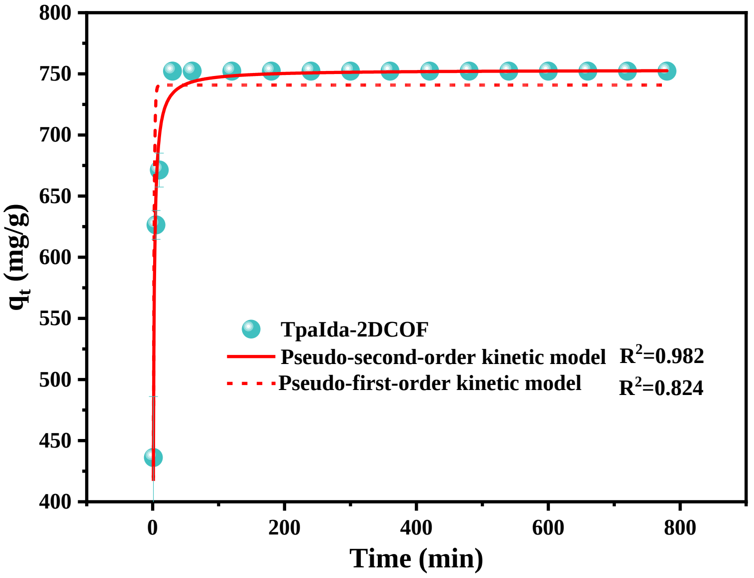

Figure S14. The fitting results of Pseudo-first-order model and pseudo-second-order model for TpaIda-2DCOF.

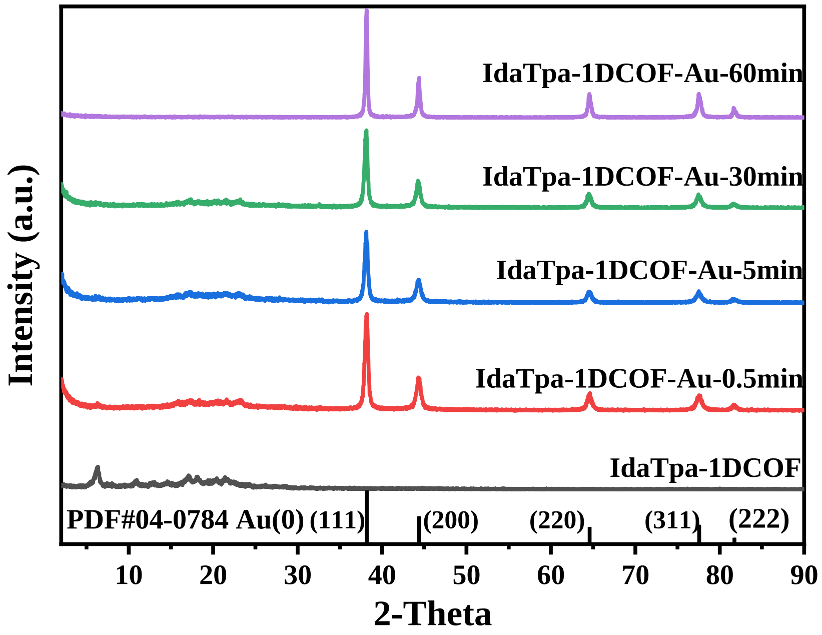

Figure S15. PXRD pattern before and after adsorption.

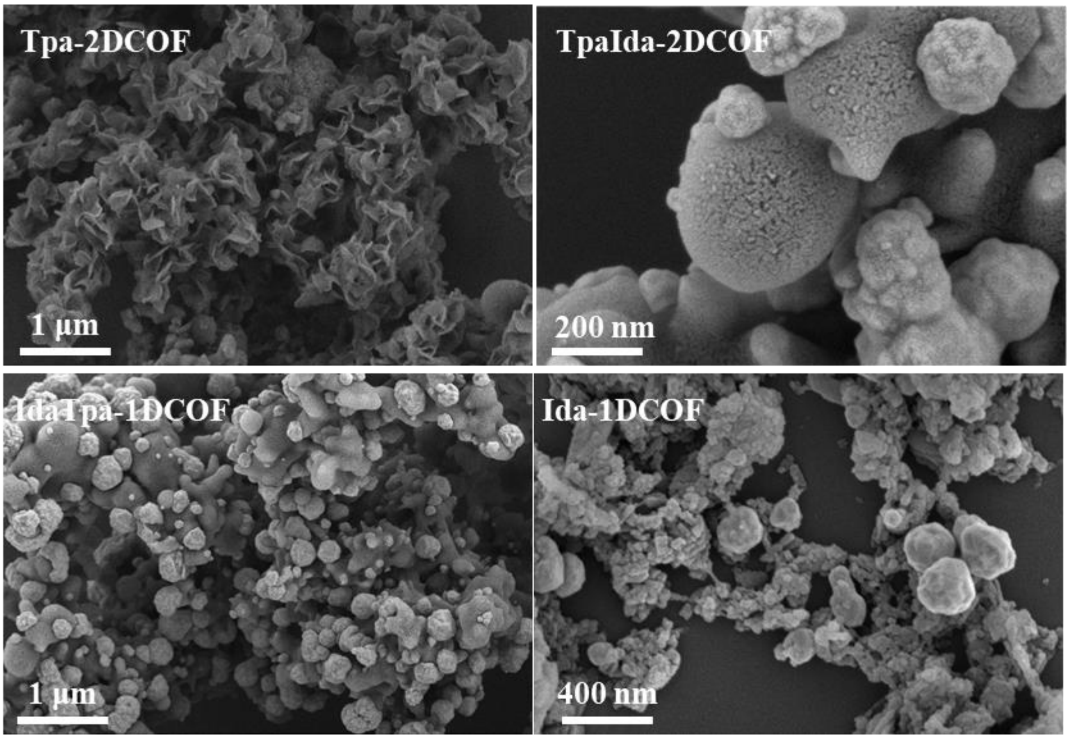

Figure S16. The SEM imaging of Tpa-2DCOF, TpaIda-2DCOF, IdaTpa-1DCOF and Ida-1DCOF after adsorption.

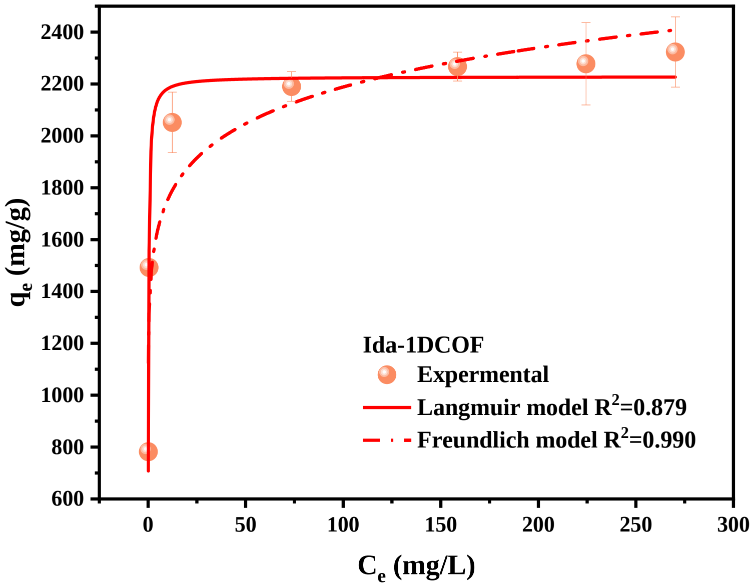

Figure S17. The fitting results of Langmuir model and Freundlich model for Ida-1DCOF.

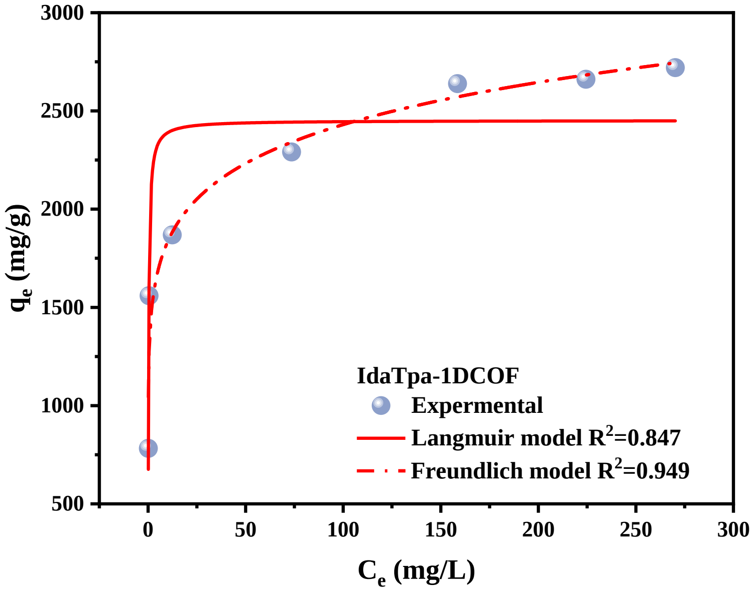

Figure S18. The fitting results of Langmuir model and Freundlich model for IdaTpa-1DCOF.

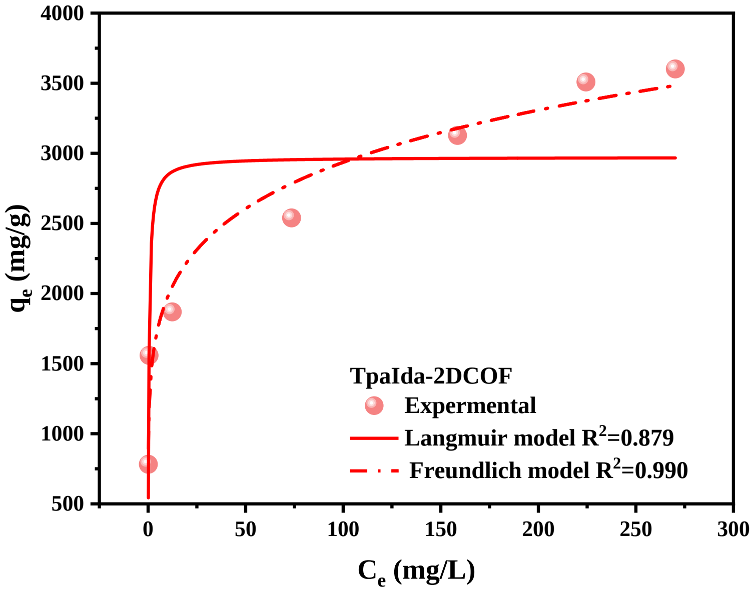

Figure S19. The fitting results of Langmuir model and Freundlich model for TpaIda-2DCOF.

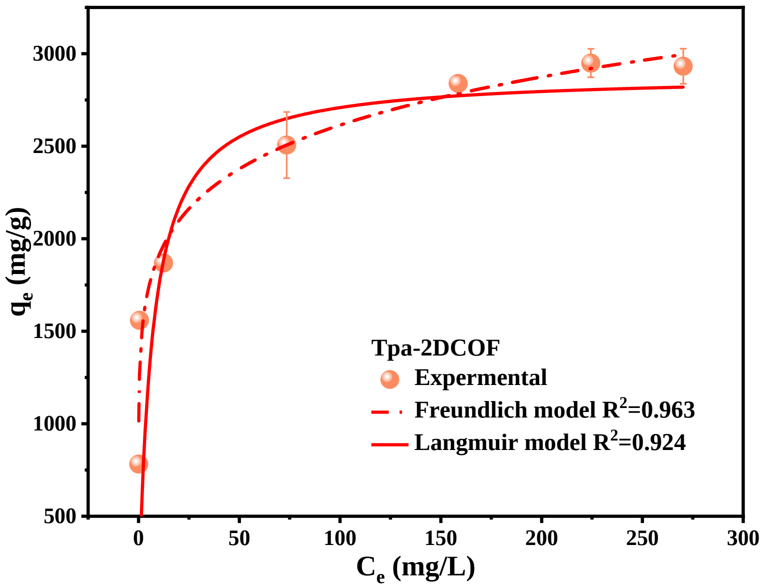

Figure S20. The fitting results of Langmuir model and Freundlich model for Tda-2DCOF.

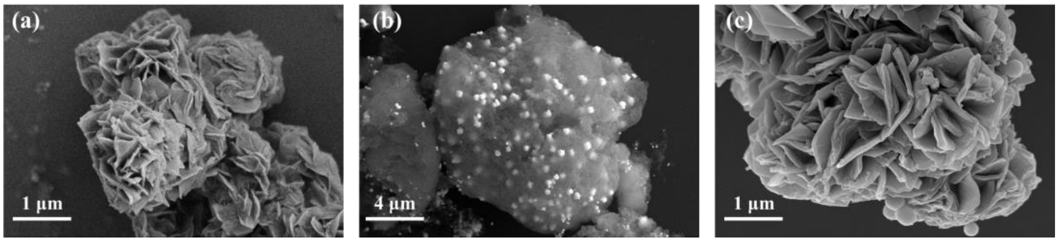

Figure S21. The SEM of Tpa-2DCOF(a), Tpa-2DCOF-Au before (b) and after(c) sonication.

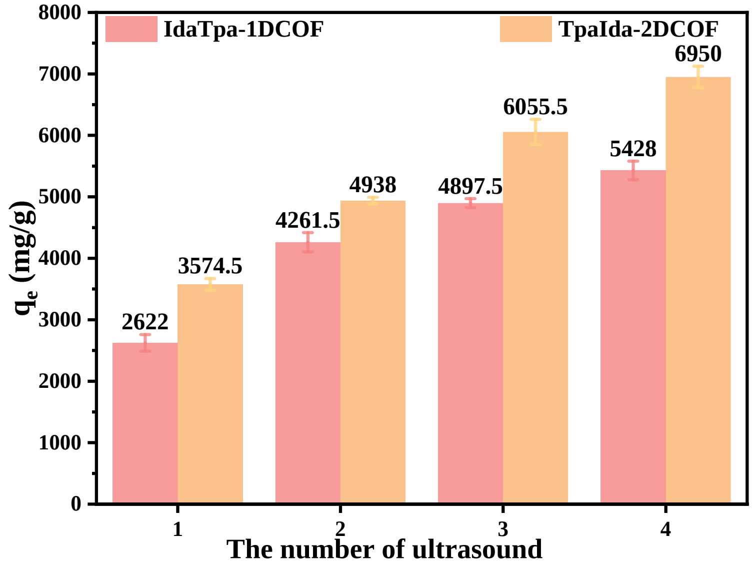

Figure S22. Ultrasound-assisted peeling experiment. Error bars represent the standard error of the mean.

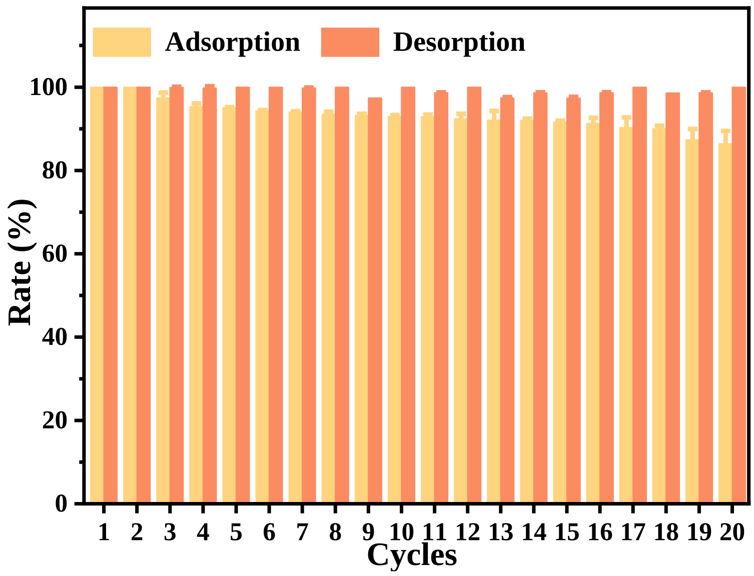

Figure S23. The Au(III) removal efficiency of Tpa-2DCOF for 20 cycles. Error bars represent the standard error of the mean.

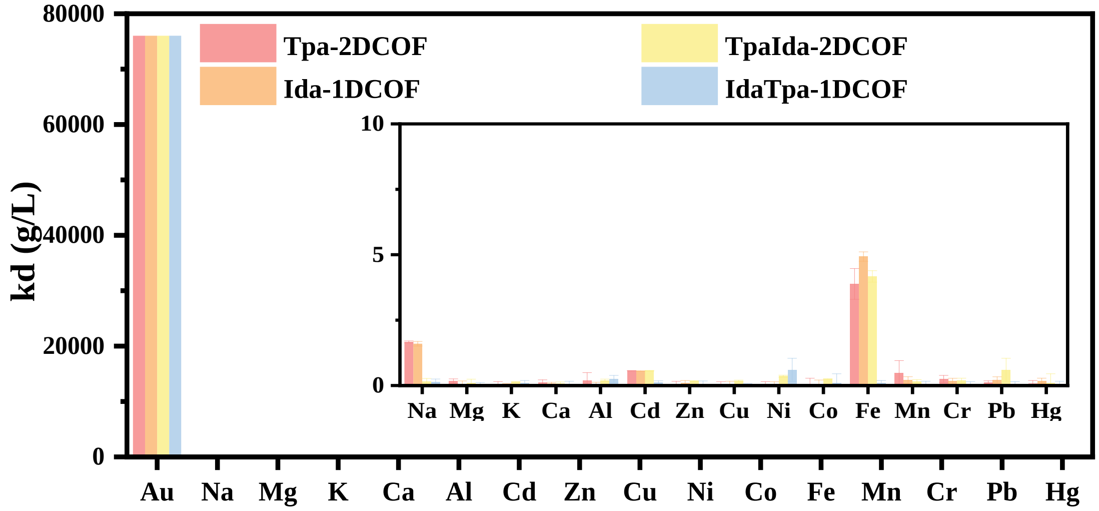

Figure S24. The distribution coefficients of different ions. Error bars represent the standard error of the mean.

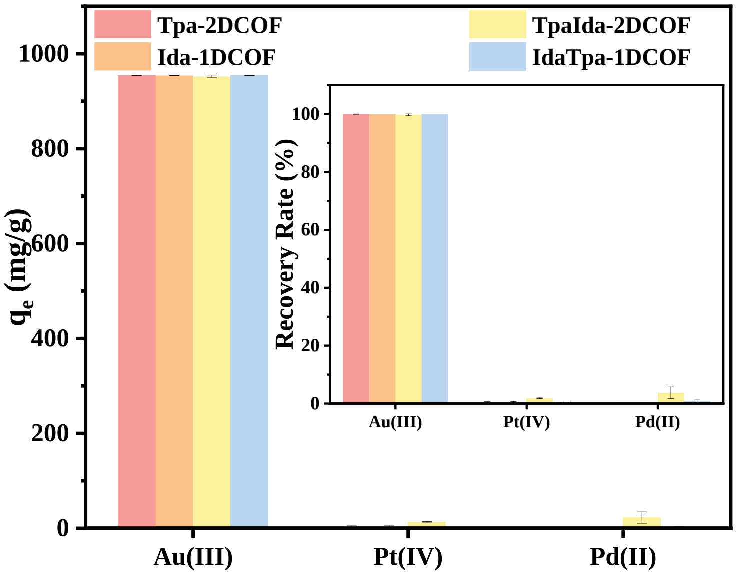

Figure S25. Effect of Pt(IV) and Pd(II) on Au(III) adsorption. Error bars represent the standard error of the mean.

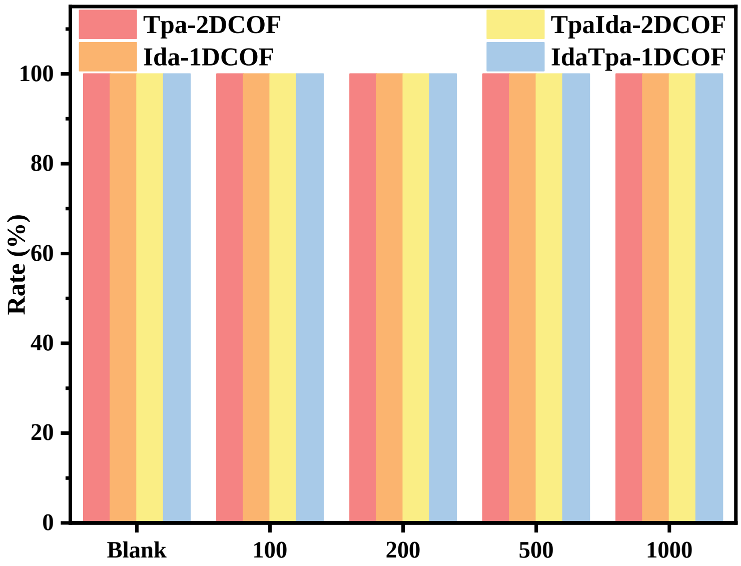

Figure S26. Effect of ionic strength on Au(III) adsorption. Error bars represent the standard error of the mean.

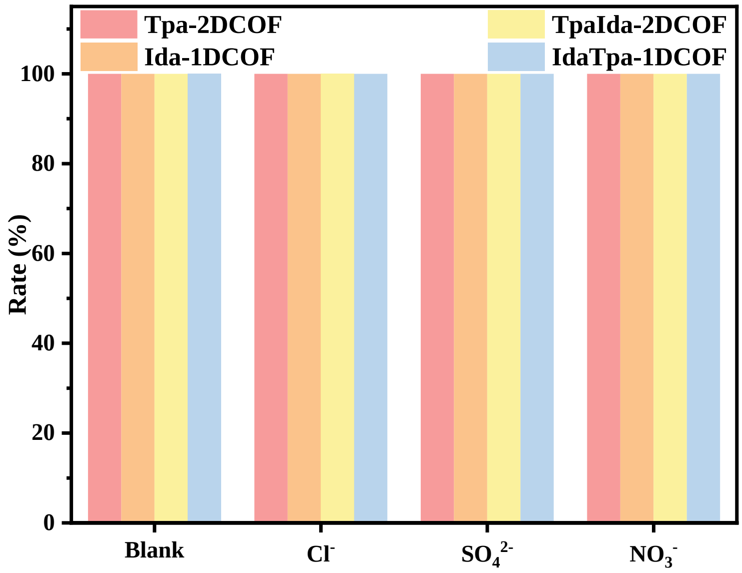

Figure S27. Effects of different anions on Au(III) adsorption. Error bars represent the standard error of the mean.

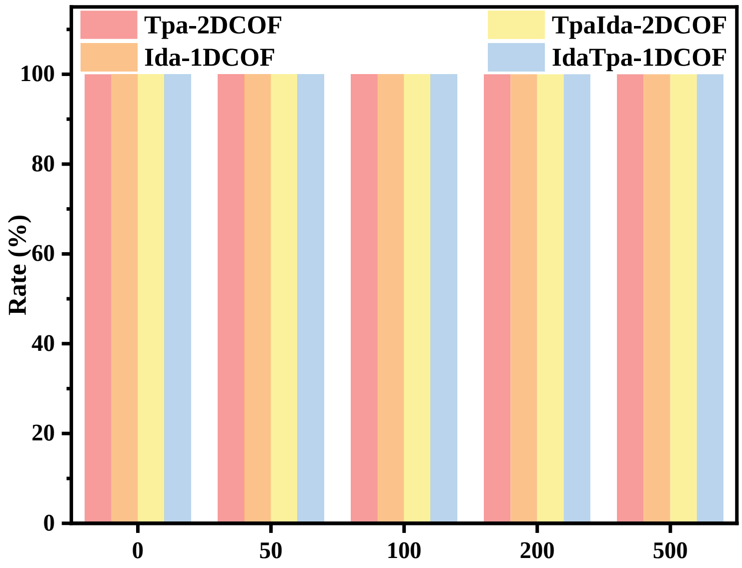

Figure S28. Effect of different concentrations of Cu(II), Ni(II) and Zn(II) on Au(III) adsorption. Error bars represent the standard error of the mean.

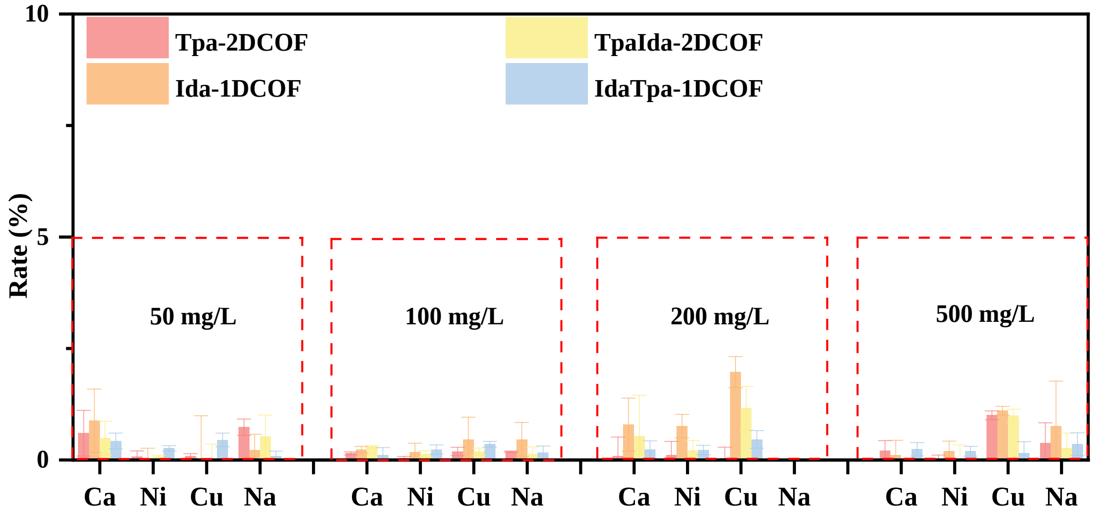

Figure S29. Removal of Cu(II), Ni(II) and Zn(II) at different concentrations. Error bars represent the standard error of the mean.

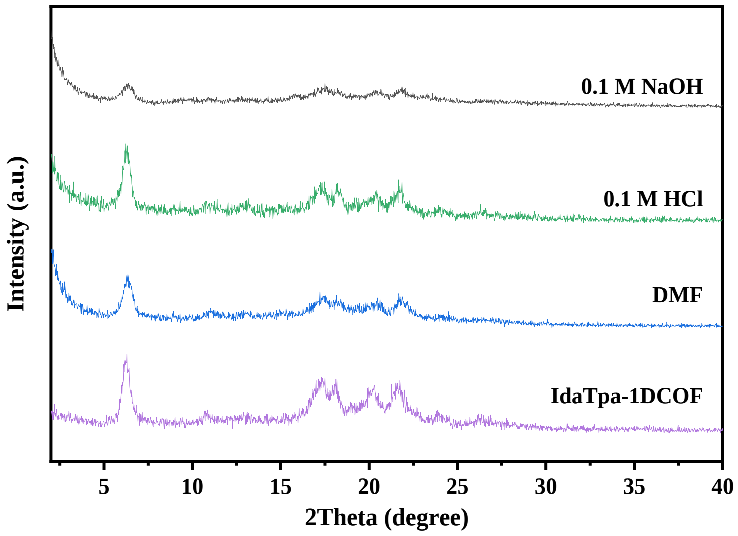

Figure S30. PXRD patterns of IdaTpa-1DCOF before and after treatment under DMF, 0.1 M HCl and 0.1 M KOH for 1 days.

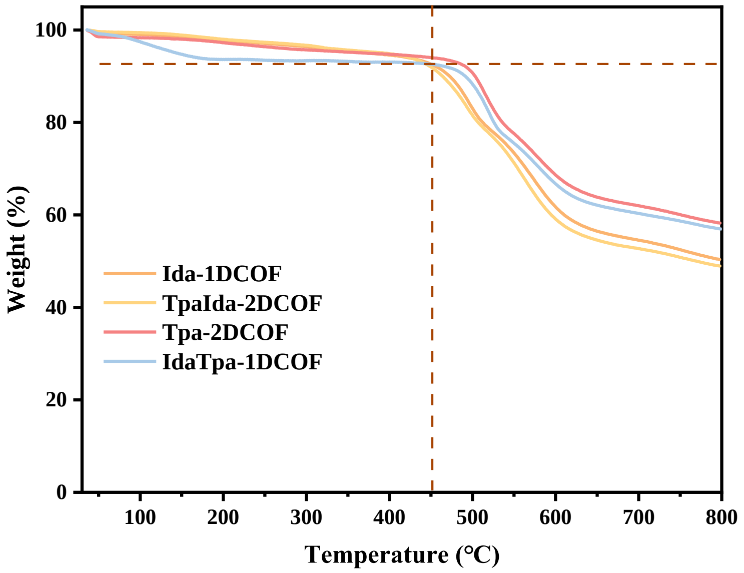

Figure S31. TGA curve of the Tpa-2DCOF, Ida-1DCOF, IdaTpa-1DCOF and TpaIda-2DCOF.

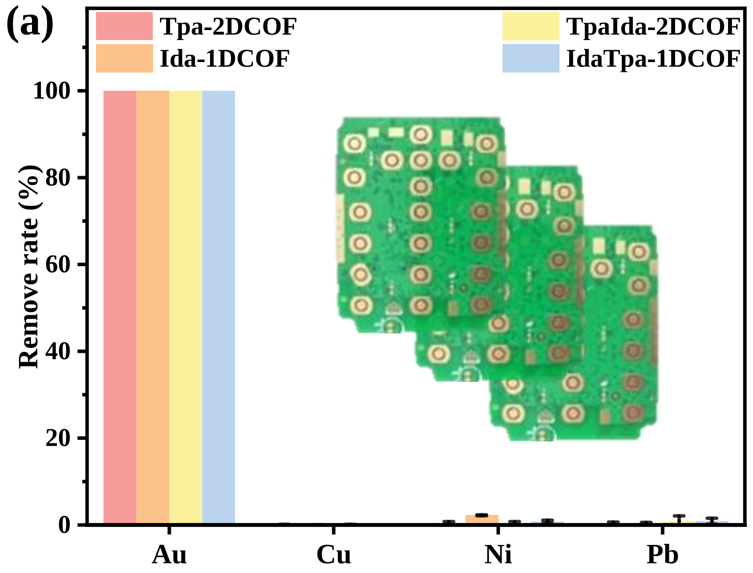

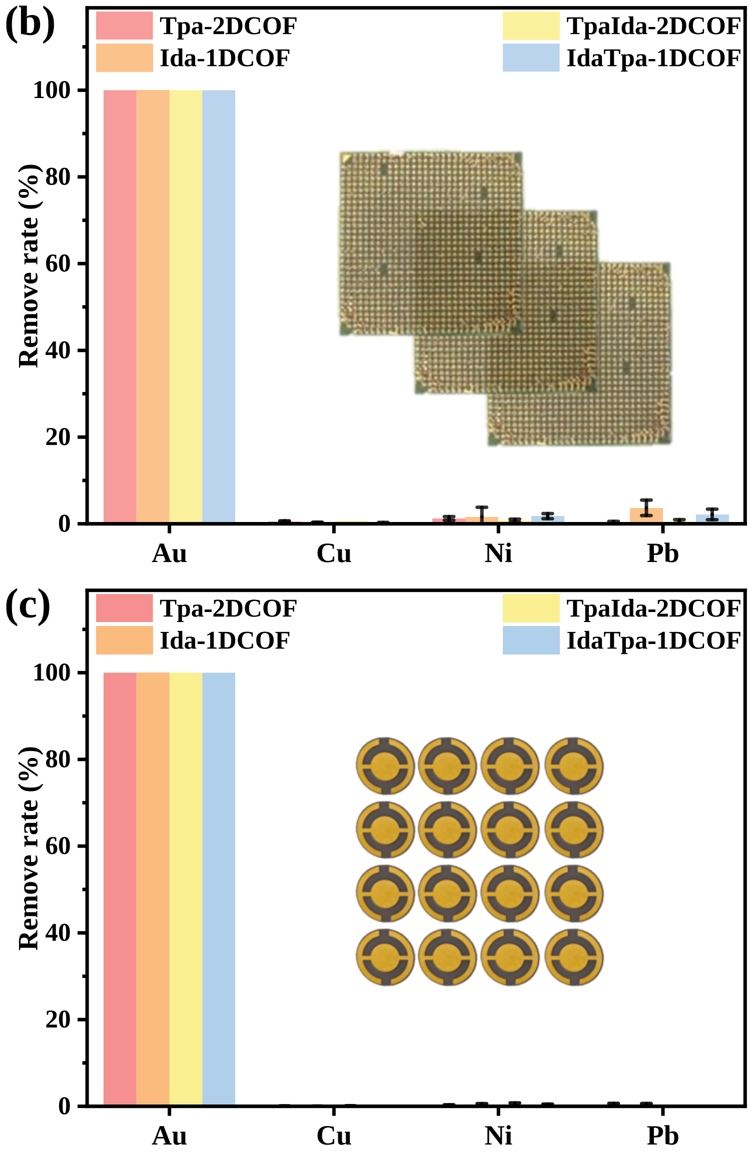

Figure S32. Adsorption performance of four COFs in (a) PCB leaching solution, (b) CPU leaching solution and (c) INFICON quartz chips.

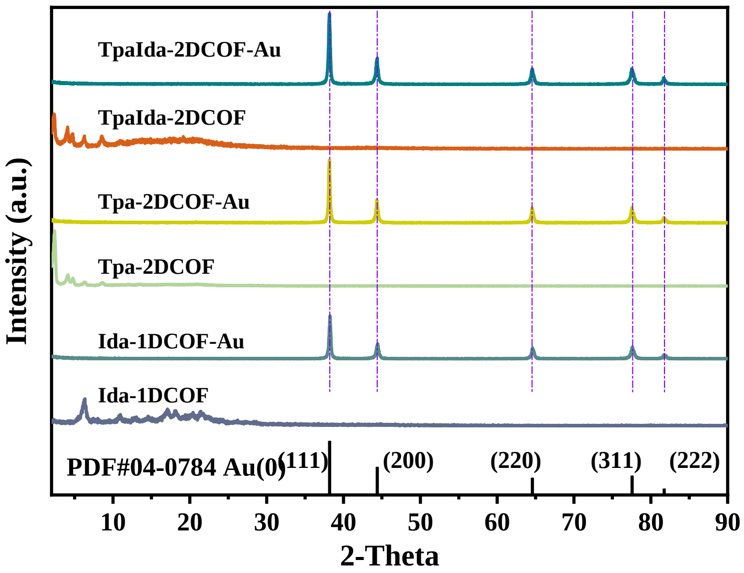

Figure S33. PXRD patterns of different COFs after the adsorption process.

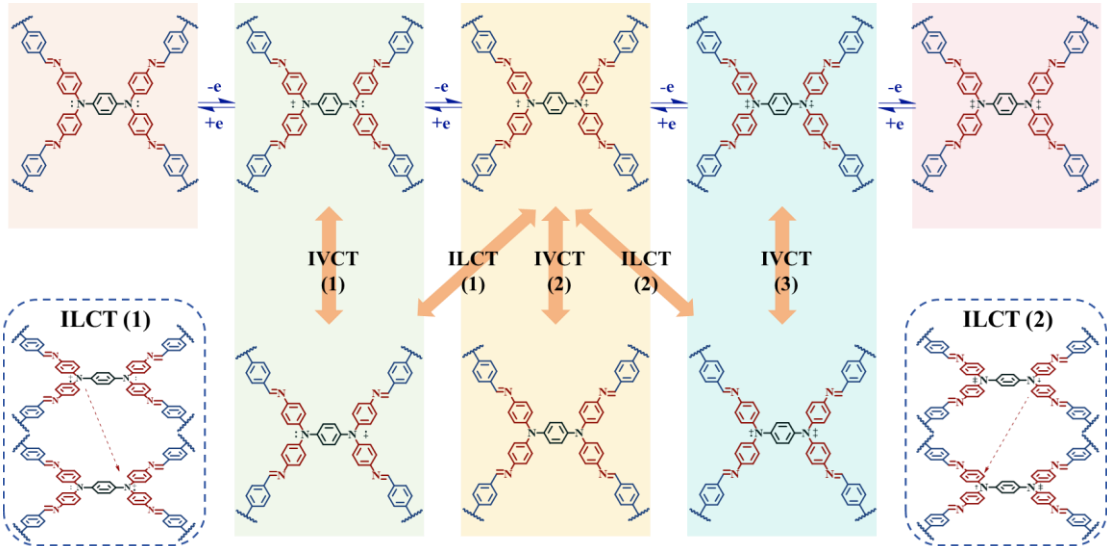

Figure S34. Schematic illustration of the proposed oxidation/reduction pathway of Tpa-2DCOF (IVCT: intervalence charge transfer, ILCT: interlayer charge transfer).

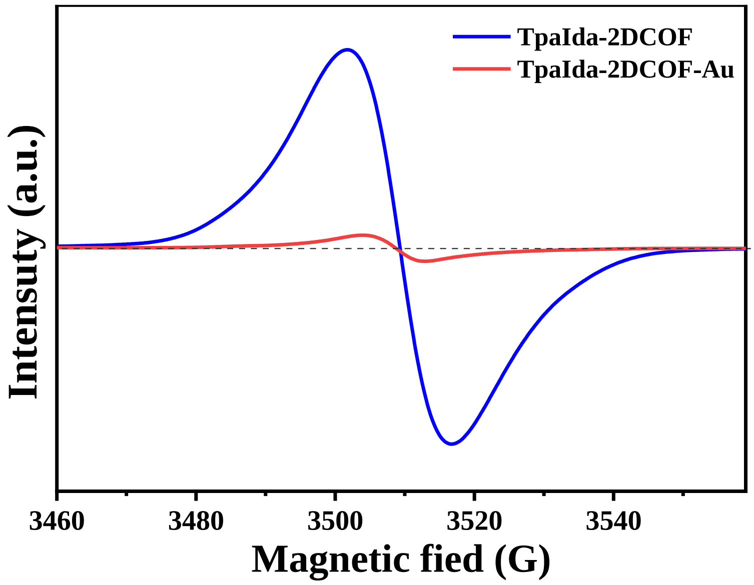

Figure S35. Electron paramagnetic resonance spectroscopy of TpaIda - 2DCOF. (Blue line) Before adsorption; (Red line) After adsorption.

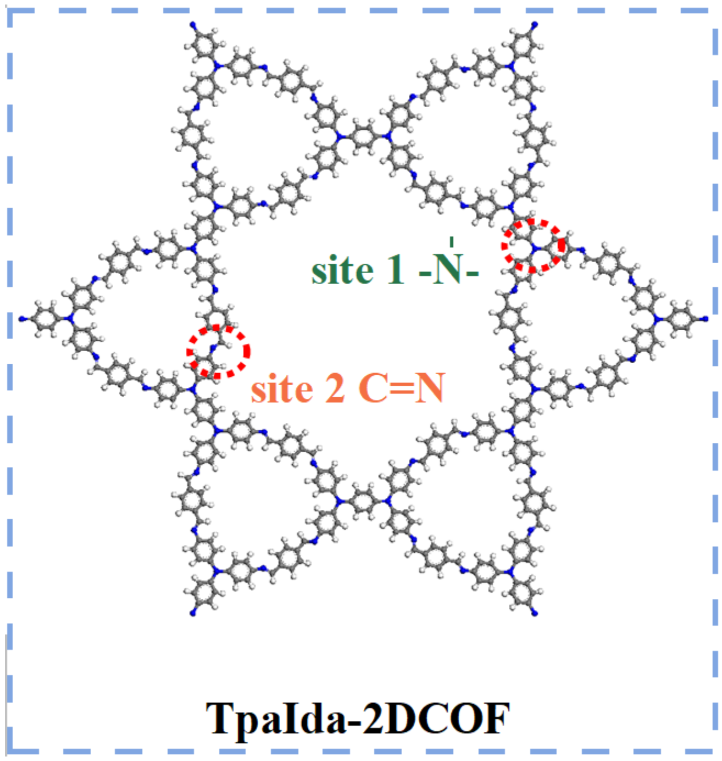

Figure S36. Distribution diagram of active sites.

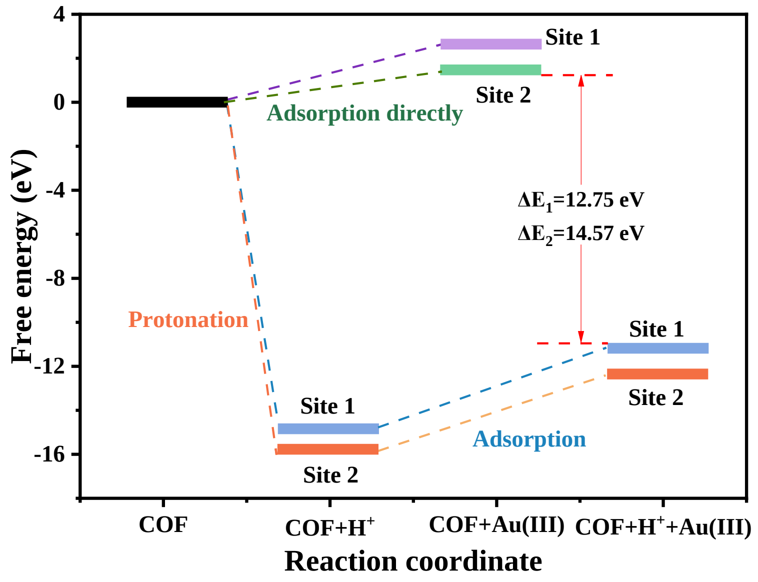

Figure S37. The reaction coordinate for different adsorption sites.

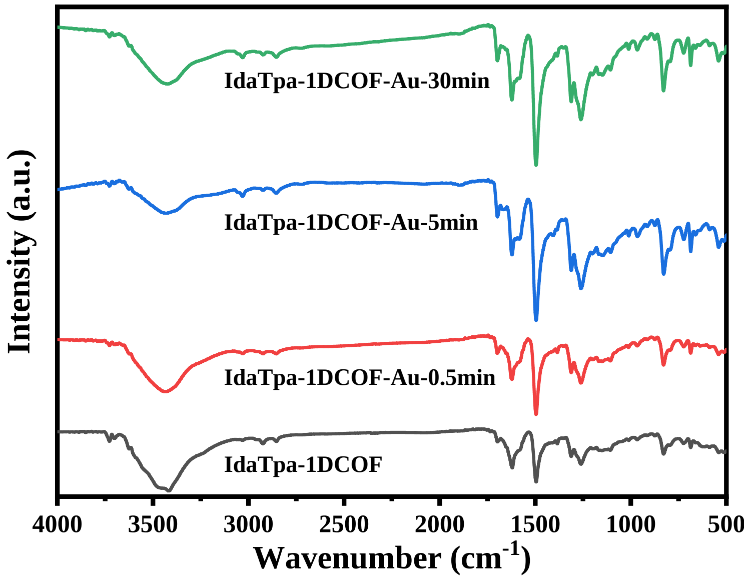

Figure S38. FT - IR spectra of IdaTpa - 1DCOF during Au(III) adsorption at different times.

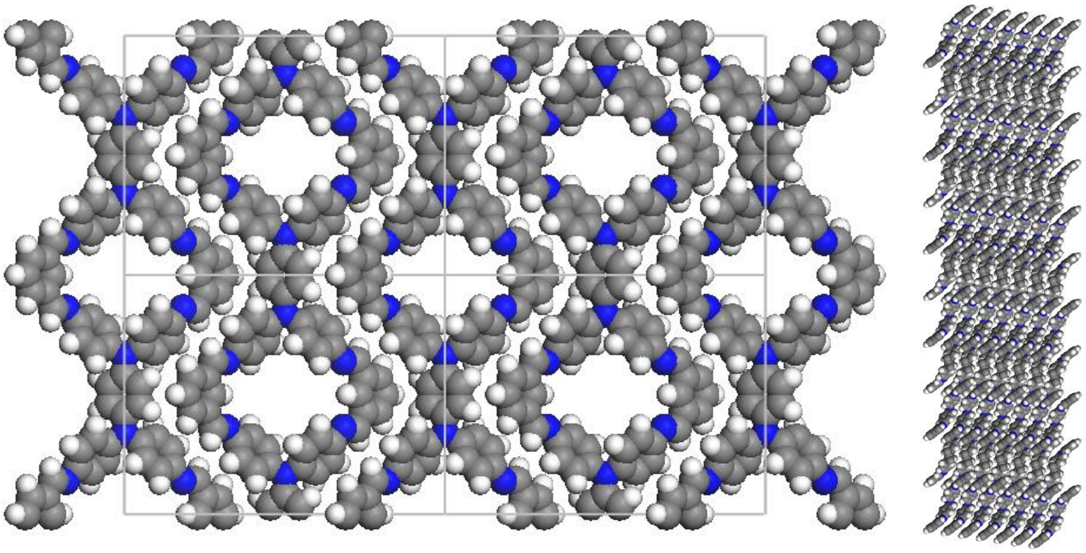

Figure S39. Top view and side-view projected along its c and b axis of the crystal lattices for Ida-1DCOF with sql-staggered stacking models.

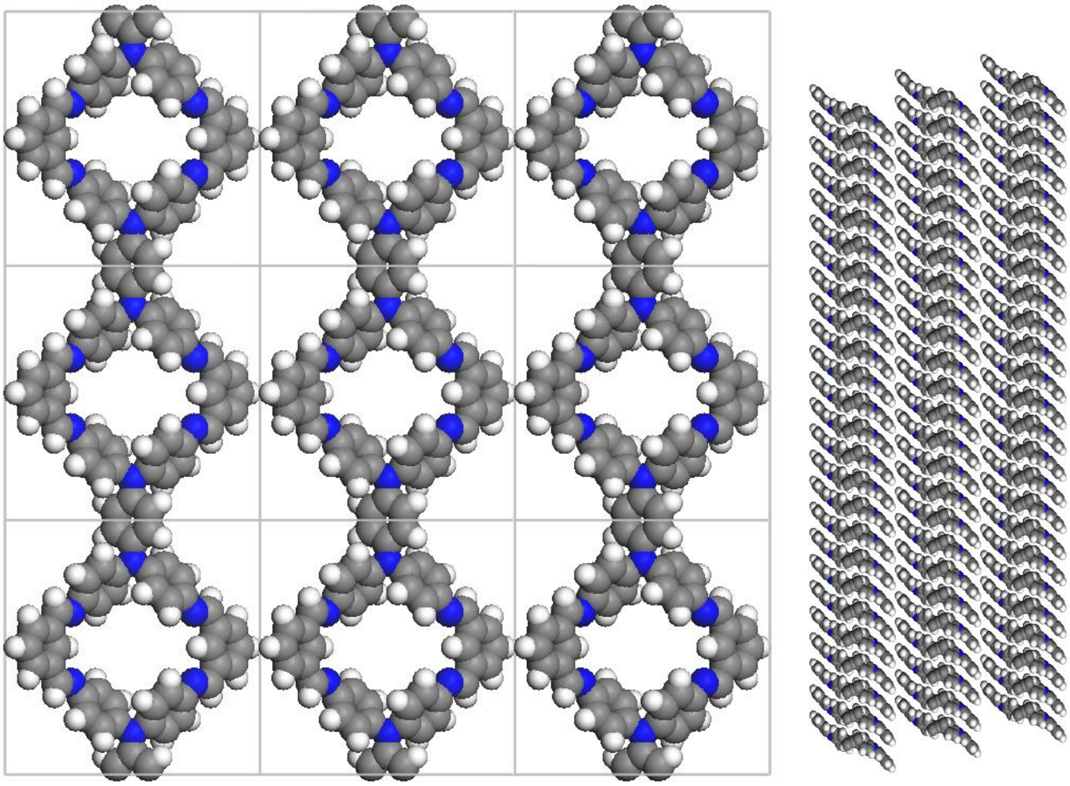

Figure S40. Top view and side-view projected along its c and b axis of the crystal lattices for Ida-1DCOF with sql-parallel stacking modes.

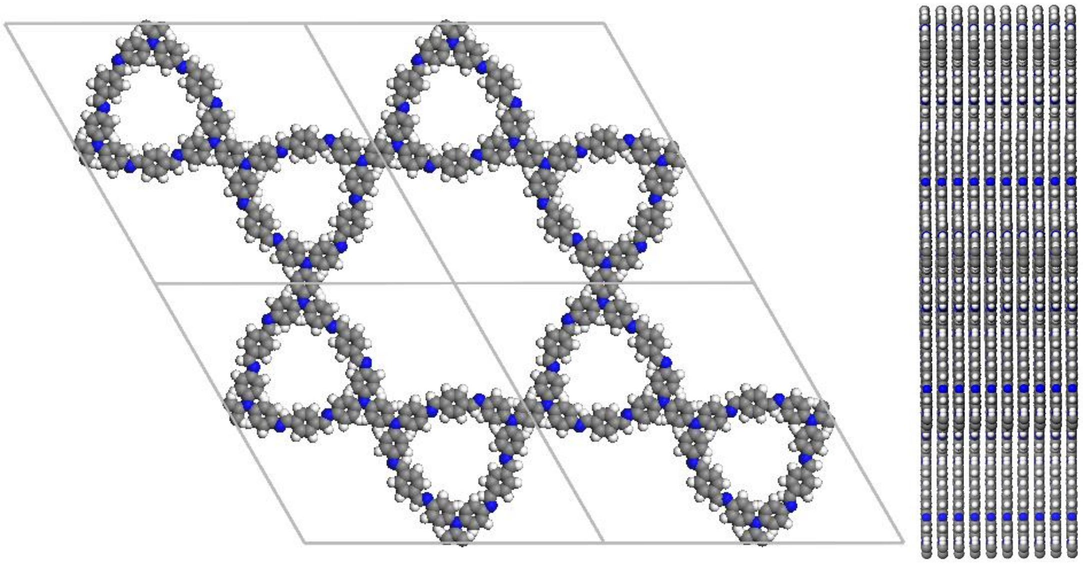

Figure S41. Top view and side-view projected along its c and b axis of the crystal lattices for Tpa-2DCOF with kgm-AA stacking models.

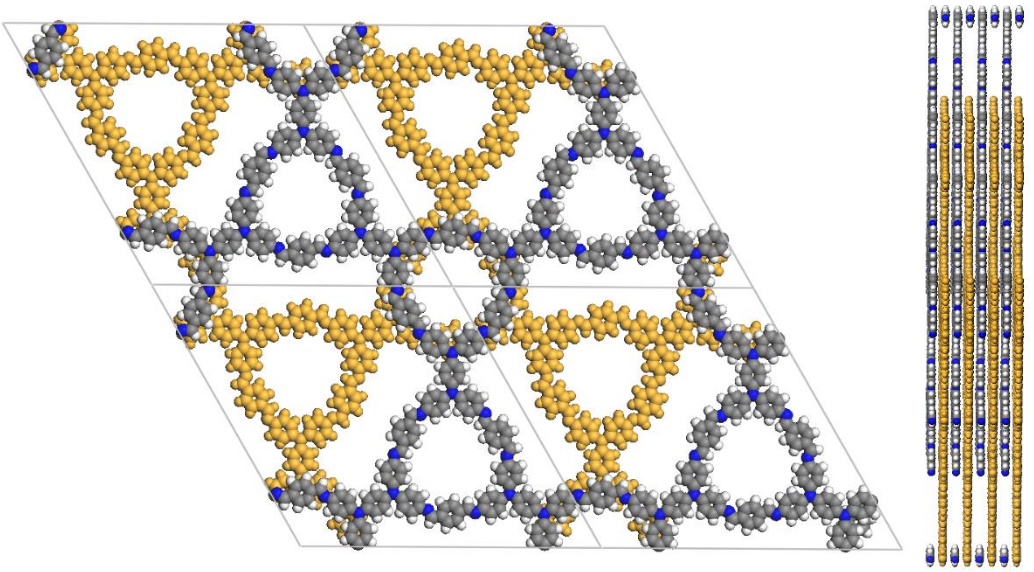

Figure S42. Top view and side-view projected along its c and b axis of the crystal lattices for Tpa-2DCOF with kgm-AB stacking models.

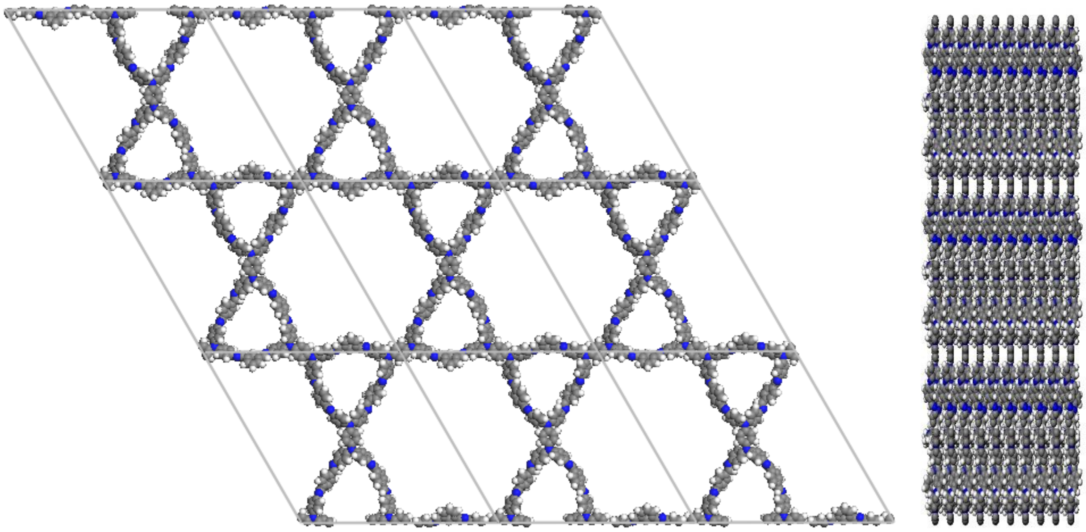

Figure S43. Top view and side-view projected along its c and b axis of the crystal lattices for TpaIda-2DCOF with kgm-AA stacking models.

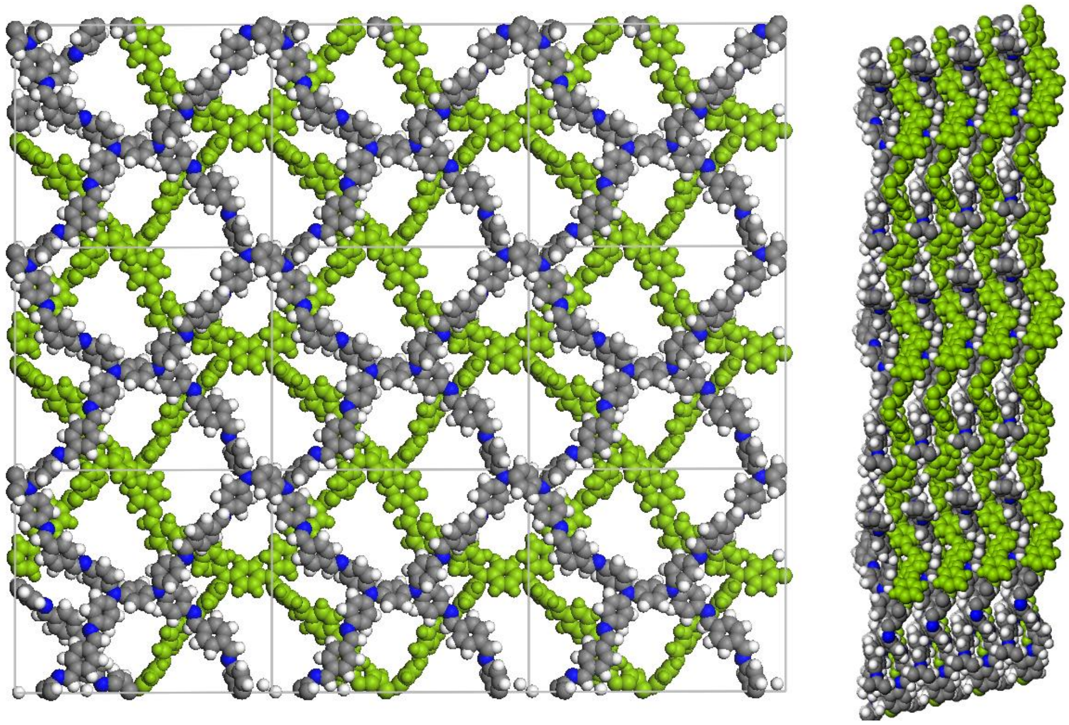

Figure S44. Top view and side-view projected along its c and b axis of the crystal lattices for TpaIda-2DCOF with kgm-AB stacking models.

Table S1. Elemental analysis.

	Theoretical value (%)			Analysis value (%)		
	C	N	H	C	N	H
**Tpa-2DCO**F ($C_{138}N_{18}H_{118}$)	81.7	12.4	5.8	75.4	10.6	4.8
**Ida-1DCOF** ($C_{92}N_{12}H_{64}$)	82.5	12.6	4.8	72.9	9.2	4.6
**IdaTpa-1DCOF(**$C_{92}N_{12}H_{64}$**)**	82.5	12.6	4.8	73.5	9.8	4.5
**TpaIda-2DCOF** ($C_{138}N_{18}H_{118}$)	81.7	12.4	5.8	75.8	10.5	4.9

Table S2. Transferred electrons between fragments.

**Fragments**†	**Electron number**	**Fragments**	**Electron number**
**1→2**	0.022	2→1	0.027
**1→3**	0.013	3→1	0.299
**2→3**	0.015	3→2	0.281

Notes: Fragment 1 represents phenyl, fragment 2 represents imine bond, fragment 2 represents triphenylamine functional group.

Intrinsic charge transfer percentage, CT(%): 65.966 %

Intrinsic local excitation percentage, LE(%): 21.884 %

Table S3. Hall effect

**Sample**	**T (K)**	**Resistivity (ohm-cm)**	**Hall mobility (cm^2 $V^{-1}S^{-1}$)**	**Carrier concentration (1/cm^{-3})**	**Hall effect (cm^3/C)**	**f-factor**
**Ida-1DCOF**	300	56960	0.923	1.18678E+14	-52598	0.967
**IdaTpa-1DCOF**		20190	1.139	2.714E+14	-23000	0.980
**Tpa-2DCOF**		16860	1.290	2.86787E+14	-21766	0.995
**TpaIda-2DCOF**		9660	1.599	4.04026E+14	-15450	0.925

Table S4. Summary of the intrinsic charge mobilities of COF materials at ambient temperature reported in literature.

Sample	Hall mobility ($cm^2\ V^{-1}S^{-1}$)	Ref.	Sample	Hall mobility ($cm^2\ V^{-1}S^{-1}$)	Ref.
**CuPc-pz COF**	0.9±0.2	6	**TTF-Ph-COF**	0.2	7
**NiPc COF**	1.3	8	**TTF-Py-COF**	0.008	
**HBC-COF**	0.7	9	**2D D-A COF**	0.01	10
**ZnP-COF**	0.032	11	**IdaTpa-1DCOF**	1.139	This work
**Tpa-2DCOF**	1.291	This work	**TpaIda-2DCOF**	1.599	This work

Table S5. Thermodynamic parameters.

Sample	ΔG^0 (KJ mol^{-1})				ΔH^0 (KJ mol^{-1})	ΔS^0 (J $(mol\cdot K)^{-1}$)
	288K	293K	298K	303K		
**Tpa-2DCOF**	-9.070	-10.529	-11.988	-13.447	74.975	291.821
**TpaIda-2DCOF**	-9.223	-11.060	-12.898	-14.735	96.611	367.479
**IdaTpa-1DCOF**	-7.414	-8.462	-9.510	-10.557	52.925	209.513
**Ida-1DCOF**	-6.679	-8.071	-9.464	-10.856	73.535	278.519

Table S6. Kinetic parameters.

Material	Pseudo-first-order kinetic model			Pseudo-second-order kinetic model		
	q_e (mg g^{-1})	k_1 (min)	R^2	q_e (mg g^{-1})	k_2 (min)	R^2
**Tpa-2DCOF**	740.4	0.615	0.842	754.3	0.00126	0.979
**TpaIda-2DCOF**	740.8	0.828	0.824	753.3	0.00167	0.982
**Ida-1DCOF**	743.4	0.187	0.880	757.0	0.000462	0.993
**IdaTpa-1DCOF**	748.4	0.229	0.919	758.4	0.000658	0.989

Table S7. Isotherm parameters.

Isotherm type	Parameters	Tpa-2DCOF	TpaIda-2DCOF	IdaTpa-1DCOF	Ida-1DCOF
**Langmuir**	q_m (mg g^{-1})	2888	2972	2451	2228
	K_L (L mg^{-1})	0.151	2.234	3.805	4.655
	R^2	0.770	0.714	0.847	0.879
**Freundlich**	k_F (mg g^{-1})(L mg^{-1}) n^{-1}	1391	1328	1383	1404
	n	-0.137	-0.172	-0.122	-0.096
	R^2	0.962	0.958	0.948	0.978

Table S8. Comparison of adsorption capacity and cost of different materials.

Adsorbent	Cost† (CNY g^{-1})	pH	q_m (mg g^{-1})	Dose (g L^{-1})	Kinetic Initial Concentration (mg L^{-1})	Saturation Time (min)	References
**Ionic-COF-Br**	762	2.5-4.5	793	0.4	N.A.†	N.A.†	12
**Ionic-COF-AcO**	762	2.5-4.5	757				
**Ionic-COF-Cl**	762	2.5-4.5	1270	0.4	139	40	
**TzDa-COF**	559	2.0-5.0	1866	1.0	250	30	13
**COF-pPDI@TAP**	35	2.0-8.0	779	1.0	300	360	14
**MTpPa-1**	251	1.0-7.0	1737	0.5	200	480	15
**COF-V-DTT**	1291	1.0-6.0	652	0.05	10	120	16
**COF-V-S-β-CD**	2740	1.0-6.0	820				
**TY-Hz COF**	447	1.0-3.0	1008	0.2	300	2	17
**JNU-1**	149	N.A.†	1124	1.0	396	0.17	18
**PYTA-PATA-COF**	4901	2.0 - 12.0	1774	0.1	25	30	19
**PYTA-BDTA-COF**	1499	2.0 - 12.0	1752				
**PYTA-TDTA-COF**	2246	2.0 - 12.0	1880				
**COF-HNU25**	1516	1.0-7.0	1725	1.0	200	20	20
**COF-HNU26**	1618	1.0-7.0	1362				
**TpDa-COF**	830	3.0-8.0	982	0.1	100	360	21
**JNM-100**	N.A.†	1.0-10.0	708	1.0	100	5	22
**JNM-100-AO**	N.A.†	1.0-10.0	954			3	
**PYTA-PZDH-COF**	4100	2.0- 12.0	2314	0.1	25	10	23
**PYTA-BPDH-COF**	1430	2.0 - 12.0	1810			30	
**Tp-BTD-AA**	790	2.0-9.0	1706	0.2	100	>360	24
**Tp-BTD-AB**	790	2.0-9.0	460				
**Tp-BTD-ABC**	790	2.0-9.0	622				
**TpTsc COF**	352	1.0-7.0	4400	0.17	300	3000	25
**TbTsc COF**	382	1.0-7.0	1071	0.17	300	1500	
**BMTA-TFPM-COF**	5838	2.0-12.0	570	0.2	30	30	26
**COP**	551	0.5-6.0	1945	1.0	3000	720	27
**Ida-1DCOF**	10	1.0-11.0	2323	0.1	100	60	This work
**Tpa-2DCOF**	10	1.0-11.0	2932	0.1	100	30	
**IdaTpa-1DCOF**	10	1.0-11.0	2720	0.1	100	30	
**TpaIda-2DCOF**	10	1.0-11.0	3601	0.1	100	30	

Notes: N.A.† = not available; Cost† = the approximate cost of the monomer, the data is from https://www.tansoole.com.

Supplementary references

1. M. J. Frisch, G. W. Trucks, H. B. Schlegel, G. E. Scuseria, M. A. Robb, J. R. Cheeseman, G. Scalmani, V. Barone, G. A. Petersson, H. Nakatsuji, X. Li, M. Caricato, A. V. Marenich, J. Bloino, B. G. Janesko, R. Gomperts, B. Mennucci, H. P. Hratchian, J. V. Ortiz, A. F. Izmaylov, J. L. Sonnenberg, Williams, F. Ding, F. Lipparini, F. Egidi, J. Goings, B. Peng, A. Petrone, T. Henderson, D. Ranasinghe, V. G. Zakrzewski, J. Gao, N. Rega, G. Zheng, W. Liang, M. Hada, M. Ehara, K. Toyota, R. Fukuda, J. Hasegawa, M. Ishida, T. Nakajima, Y. Honda, O. Kitao, H. Nakai, T. Vreven, K. Throssell, J. A. Montgomery Jr., J. E. Peralta, F. Ogliaro, M. J. Bearpark, J. J. Heyd, E. N. Brothers, K. N. Kudin, V. N. Staroverov, T. A. Keith, R. Kobayashi, J. Normand, K. Raghavachari, A. P. Rendell, J. C. Burant, S. S. Iyengar, J. Tomasi, M. Cossi, J. M. Millam, M. Klene, C. Adamo, R. Cammi, J. W. Ochterski, R. L. Martin, K. Morokuma, O. Farkas, J. B. Foresman and D. J. Fox, Gaussian 16 Rev. C.01.*Journal*, 2016.
2. B. Swain, J. Jeong, S.-k. Kim and J.-c. Lee, Separation of platinum and palladium from chloride solution by solvent extraction using Alamine 300, *Hydrometallurgy*, 2010, **104**, 1-7.
3. T. Lu and F. Chen, Multiwfn: A multifunctional wavefunction analyzer, *J. Comput. Chem.*, 2012, **33**, 580-592.
4. W. Humphrey, A. Dalke and K. Schulten, VMD: Visual molecular dynamics, *J. Mol. Graph*, 1996, **14**, 33-38.
5. Y. Li, X. Wang, Q. Xiao and X. Zhang, Study on Selective Removal of Impurity Iron from Leached Copper-Bearing Solution Using a Chelating Resin, *Minerals*, 2016, **6**.
6. M. Wang, M. Ballabio, M. Wang, H.-H. Lin, B. P. Biswal, X. Han, S. Paasch, E. Brunner, P. Liu, M. Chen, M. Bonn, T. Heine, S. Zhou, E. Cánovas, R. Dong and X. Feng, Unveiling Electronic Properties in Metal–Phthalocyanine-Based Pyrazine-Linked Conjugated Two-Dimensional Covalent Organic Frameworks, *J. Am. Chem. Soc.*, 2019, **141**, 16810-16816.
7. S. Jin, T. Sakurai, T. Kowalczyk, S. Dalapati, F. Xu, H. Wei, X. Chen, J. Gao, S. Seki, S. Irle and D. Jiang, Two-dimensional tetrathiafulvalene covalent organic frameworks: towards latticed conductive organic salts, *Chem.Eur. J.*, 2014, **20**, 14608-14613.
8. X. Ding, J. Guo, X. Feng, Y. Honsho, J. Guo, S. Seki, P. Maitarad, A. Saeki, S. Nagase and D. Jiang, Synthesis of Metallophthalocyanine Covalent Organic Frameworks That Exhibit High Carrier Mobility and Photoconductivity, *Angew. Chem. Int. Ed.*, 2011, **50**, 1289-1293.
9. S. Dalapati, M. Addicoat, S. Jin, T. Sakurai, J. Gao, H. Xu, S. Irle, S. Seki and D. Jiang, Rational design of crystalline supermicroporous covalent organic frameworks with triangular topologies, *Nat Commun.*, 2015, **6**, 7786.
10. X. Feng, L. Chen, Y. Honsho, O. Saengsawang, L. Liu, L. Wang, A. Saeki, S. Irle, S. Seki, Y. Dong and D. Jiang, An Ambipolar Conducting Covalent Organic Framework with Self-Sorted and Periodic Electron Donor-Acceptor Ordering, *Adv. Mater*, 2012, **24**, 3026-3031.
11. X. Feng, L. Liu, Y. Honsho, A. Saeki, S. Seki, S. Irle, Y. Dong, A. Nagai and D.

Jiang, High-Rate Charge-Carrier Transport in Porphyrin Covalent Organic Frameworks: Switching from Hole to Electron to Ambipolar Conduction, *Angew. Chem. Int. Ed.*, 2012, **51**, 2618-2622.
12. J. Zhao, Z. Qiao, Y. He, R. Zhang, H. Li, X. Song, D. Cao and S. Wang, Anion-Regulated Ionic Covalent Organic Frameworks for Highly Selective Recovery of Gold from E-Waste, *Angew. Chem. Int. Ed.* , 2025, **64**, e202414366.
13. S. Zhong, Y. Wang, T. Bo, J. Lan, Z. Zhang, L. Sheng, J. Peng, L. Zhao, L. Yuan, M. Zhai and W. Shi, Efficient and selective gold recovery from e-waste by simple and easily synthesized covalent organic framework, *Chem. Eng. J.*, 2023, **455**, 140523.
14. D. Xiang, M. Zhu, Y. Chen, S. Wang, Z. Peng, D. Zhang and L. Fu, Design and synthesis of covalent organic framework based on 2,4,6-triaminopyrimidine for efficient capture of Au(III) ion, *J. Mol. Liq*, 2024, **405**, 125054.
15. Y. Bai, J. Yang, Q. Shuai and L. Huang, Highly efficiency and selective recovery of gold using magnetic covalent organic framework through synergistic adsorption and reduction, *Colloida Surf., A*, 2023, **657**, 130593.
16. F. Liu, S. Wang, Z. Hu and B. Hu, Post-synthetically functionalized covalent organic frameworks for highly efficient recovery of gold from leaching liquor of electronic waste, *Sep. Purif. Technol*, 2024, **329**, 125218.
17. L. Zhang, J.-Q. Fan, Q.-Q. Zheng, S.-J. Xiao, C.-R. Zhang, S.-M. Yi, X. Liu, W. Jiang, Q.-G. Tan, R.-P. Liang and J.-D. Qiu, A 2D mesoporous hydrazone covalent organic framework for selective detection and ultrafast recovery of Au(III) from electronic waste, *Chem. Eng. J.*, 2023, **454**, 140212.
18. H.-L. Qian, F.-L. Meng, C.-X. Yang and X.-P. Yan, Irreversible Amide-Linked Covalent Organic Framework for Selective and Ultrafast Gold Recovery, *Angew. Chem. Int. Ed.*, 2020, **59**, 17607-17613.
19. M. Liu, D. Jiang, Y. Fu, G. Zheng Chen, S. Bi, X. Ding, J. He, B.-H. Han, Q. Xu and G. Zeng, Modulating Skeletons of Covalent Organic Framework for High-Efficiency Gold Recovery, *Angew. Chem. Int. Ed.*, 2024, **63**, e202317015.
20. J. Qiu, C. Xu, X. Xu, Y. Zhao, Y. Zhao, Y. Zhao and J. Wang, Porous Covalent Organic Framework Based Hydrogen-Bond Nanotrap for the Precise Recognition and Separation of Gold, *Angew. Chem. Int. Ed.*, 2023, **62**, e202300459.
21. D. Mei and B. Yan, A 2D Acridine-Based Covalent Organic Framework for Selective Detection and Efficient Extraction of Gold from Complex Aqueous-Based Matrices, *Angew. Chem. Int. Ed.*, 2024, **63**, e202402205.
22. J. Luo, X. Luo, M. Xie, H.-Z. Li, H. Duan, H.-G. Zhou, R.-J. Wei, G.-H. Ning and D. Li, Selective and rapid extraction of trace amount of gold from complex liquids with silver(I)-organic frameworks, *Nat. Commun.*, 2022, **13**, 7771.
23. X. Yang, D. Jiang, Y. Fu, X. Li, G. Liu, X. Ding, B.-H. Han, Q. Xu and G. Zeng, Synergistic Linker and Linkage of Covalent Organic Frameworks for Enhancing Gold Capture, *Small*, 2024, **20**, 2404192.
24. S. Yang, T. Li, Y. Cheng, W. Fan, L. Wang, Y. Liu, L. Bian, C.-H. Zhou, L.-Y. Zheng and Q.-E. Cao, Covalent Organic Framework Isomers for Photoenhanced Gold Recovery from E-Waste with High Efficiency and Selectivity, *Acs Sustain Chem Eng*, 2022, **10**, 9719-9731.

25. L. Zhang, Q.-Q. Zheng, S.-J. Xiao, J.-Q. Chen, W. Jiang, W.-R. Cui, G.-P. Yang, R.-P. Liang and J.-D. Qiu, Covalent organic frameworks constructed by flexible alkyl amines for efficient gold recovery from leaching solution of e-waste, *Chem. Eng. J.*, 2021, **426**, 131865.
26. M. Liu, H.-Y. Kong, S. Bi, X. Ding, G. Z. Chen, J. He, Q. Xu, B.-H. Han and G. Zeng, Non-Interpenetrated 3D Covalent Organic Framework with Dia Topology for Au Ions Capture, *Adv. Funct. Mater.* , 2023, **33**, 2302637.
27. A. Li, N. Zheng, T. Yang, J. Xie, L. Li, K. Tang and C. Zhou, Highly selective enrichment of Au using enaminone covalent organic polymers (COP), *RSC ADV*, 2021, **11**, 29807-29815.
